# LKB1 and STRADα Promote Epithelial Ovarian Cancer Spheroid Cell Invasion

**DOI:** 10.3390/cancers16223726

**Published:** 2024-11-05

**Authors:** Charles B. Trelford, Adrian Buensuceso, Emily Tomas, Yudith Ramos Valdes, Owen Hovey, Shawn Shun-Cheng Li, Trevor G. Shepherd

**Affiliations:** 1 The Mary & John Knight Translational Ovarian Cancer Research Unit, Verspeeten Family Cancer Centre, London, ON N6A 4L6, Canada; charles.trelford@gmail.com (C.B.T.);; 2 Department of Anatomy & Cell Biology, Schulich School of Medicine and Dentistry, Western University, London, ON N6A 4L6, Canada; 3 Department of Biochemistry, Schulich School of Medicine and Dentistry, Western University, London, ON N6A 4L6, Canada; 4 Children’s Health Research Institute, London, ON N6A 4L6, Canada; 5 Department of Oncology, Schulich School of Medicine and Dentistry, The University of Western Ontario, London, ON N6A 4L6, Canada; 6 Department of Obstetrics & Gynaecology, Schulich School of Medicine and Dentistry, Western University, London, ON N6A 4L6, Canada

**Keywords:** epithelial ovarian cancer (EOC), liver kinase B1 (LKB1), STE20-related kinase adaptor protein (STRAD), spheroid, organoid, mesothelial clearance, metastasis

## Abstract

Late-stage epithelial ovarian cancer (EOC) involves the widespread dissemination of malignant disease throughout the peritoneal cavity, often accompanied by ascites. EOC metastasis relies on the formation of multicellular aggregates, called spheroids. Given that Liver Kinase B1 (LKB1) is required for EOC spheroid viability and LKB1 loss in EOC cells decreases tumor burden in mice, we investigated whether the LKB1 complex controls the invasive properties of human EOC spheroids. LKB1 signalling was antagonized through the CRISPR/Cas9 genetic knockout of LKB1 and/or the RNAi-dependent targeting of STE20-related kinase adaptor protein (STRAD, an LKB1 activator). EOC spheroids expressing nuclear GFP (green) or mKate2 (red) constructs were embedded in Matrigel for real-time live-cell invasion monitoring. Migration and invasion were also assessed in spheroid culture using Transwell chambers, spheroid reattachment, and mesothelial clearance assays. The loss of LKB1 and STRAD signalling decreased cell invasion through Matrigel and Transwell membranes, as well as mesothelial cell clearance. In the absence of LKB1, zymographic assays identified a loss of matrix metalloproteinase (MMP) activity, whereas spheroid reattachment assays found that coating plates with fibronectin restored their invasive potential. A three-dimensional EOC organoid model demonstrated that organoid area was greatly reduced by LKB1 loss. Overall, our data indicated that LKB1 and STRAD facilitated EOC metastasis by promoting MMP activity and fibronectin expression. Given that LKB1 and STRAD are crucial for EOC metastasis, targeting LKB1 and/or STRAD could disrupt the dissemination of EOC, making inhibitors of the LKB1 pathway an alternative therapeutic strategy for EOC patients.

## Simple Summary

We have shown previously that Liver Kinase B1 (LKB1) expression and activity are required during ovarian cancer metastasis. Herein, we show that LKB1 and its interacting partner STRAD are required to support the invasive properties of ovarian cancer. This result was best exemplified using our in vitro organotypic model that mimics how multicellular aggregates called spheroids attach to and invade abdominal surfaces during ovarian cancer metastasis. During re-attachment and invasion, the LKB1-STRAD complex acts to produce the extracellular matrix protein fibronectin and regulates the activity of matrix metalloproteinase 9, expressed by interacting mesothelial cells. Given that both fibronectin and matrix metalloproteinase 9 activity are linked to metastasis, this provides new mechanistic insight into how LKB1 acts as a metastasis promoter in advanced ovarian cancer.

## 1. Introduction

The standard therapeutic regimen for epithelial ovarian cancer (EOC) is cytoreductive surgery combined with platinum/taxane pharmaceuticals [1]. Although most patients enter clinical remission following therapeutic intervention, more than 70% develop recurrent disease that is resistant to chemotherapy [2]. The five-year survival rate of EOC detected at the early stage is greater than 90% [3], but due to the lack of screening and asymptomatic early disease, EOC is usually diagnosed in the advanced stages [4]. Late-stage EOC has a 30% five-year survival rate, as many patients present with metastatic disease, which contributes to EOC being the most lethal gynecological malignancy in the developed world [5].

EOC has a unique mode of metastasis, in which cells disseminate from the primary tumor and form multicellular aggregates known as spheroids [6]. Compared to single cells or small cell clusters, spheroids enhance metastasis by offering resistance to hypoxia [7], anoikis [8], nutrient deprivation [9], and shear stress [10]. EOC spheroids travel through the peritoneum via peritoneal fluid and invade the mesothelium of the abdominal organs [11]. In some patients, mesothelial invasion disrupts the peritoneal lining of organs, leading to the accumulation of protein-rich fluid in the abdomen, known as malignant ascites [12]. The extracellular matrix proteins, cytokines, and growth factors present in ascites augment EOC spheroid implantation and invasion [11]. Given that spheroids are paramount for efficient EOC survival and mesothelial invasion [13], targeting spheroid formation may be an effective strategy to attenuate EOC intraperitoneal metastasis.

We have previously reported that Liver Kinase B1 (LKB1) signalling is required for EOC spheroid formation [14]. LKB1 is a ubiquitously expressed serine–threonine kinase encoded by the serine–threonine kinase 11 (*STK11*) gene, [15] which is expressed in many patient-derived ascites and immortalized EOC cell lines [14]. LKB1 functions as a heterotrimer of pseudokinase STE-20-related kinase adaptor protein (STRAD) and mouse protein 25 (MO25) [16]. Together, STRAD and MO25 regulate the nucleocytoplasmic shuttling, active site conformation, and substrate selectivity of LKB1 [17,18,19]. Although the LKB1-independent roles of STRAD and MO25 in tumorigenesis are poorly understood [20], the tumor-suppressive activity of LKB1 is well known, as inactivating *STK11* mutations are routinely detected in lung tumor biopsies [21,22].

Over the years, there has been increasing evidence of the pro-tumorigenic activity of LKB1. For instance, LKB1 activates adenosine monophosphate protein kinase (AMPK) and regulates anoikis resistance [23], reactive oxygen species scavenging [24], and autophagy [25], all augmenting tumorigenesis. To date, few studies have assessed the role of LKB1 and STRAD in EOC spheroid invasion. Given our findings that *STK11* knockout (KO) decreases EOC spheroid cell viability [14] and tumor burden in mice [26], we proposed that disrupting spheroid formation by downregulating LKB1 and STRAD would impair the metastatic properties of human EOC cells. Herein, we present evidence that LKB1 and STRAD are essential for EOC spheroid cell invasion and mesothelial clearance, as well as EOC organoid growth.

## 2. Materials and Methods

### 2.1. Cultured Cell Lines

HeyA8, OVCAR8, OVCAR3, and OVCAR4 cells were purchased from ATCC, whereas the immortalized human fallopian tube (FT190) cell line was provided by R. Drapkin (University of Pennsylvania, Philadelphia, PA, USA). The GFP-labelled ZT mesothelial cell line was provided by M. Iwanicki (Stevens Institute of Technology, Hoboken, NJ, USA). HeyA8 and OVCAR8 cell lines were cultured in RPMI1640 (Gibco), whereas OVCAR3, OVCAR4, FT190, and ZT cells were cultured in DMEM/F12 (Gibco). For all cell lines, growth medium was supplemented with 10% fetal bovine serum (FBS) and the cells were cultured in a humidified incubator with 5% carbon dioxide at 37 °C. HeyA8, HeyA8 *STK11*KO, OVCAR8, and OVCAR8 *STK11*KO cells were transduced with an IncuCyte NucLight lentiviral reagent (Sartorius) to stably express nuclear-restricted mKate2 (red) and GFP (green). Puromycin (BioShop, PUR555.2) was used to select cells expressing the mKate2 or GFP constructs. Tissue-culture-treated polystyrene (Sarstedt, Newton, NC, USA) and Ultra-Low Attachment (ULA) cluster plates (Corning, NY, USA) were used for adherent culture and spheroid culture, respectively. All cell lines were validated by short tandem repeat profiling (The Centre for Applied Genomics, The Hospital for Sick Children, Toronto, ON, Canada).

### 2.2. STK11 Knockout Cell Lines

The CRISPR/Cas9-dependent knockout of *STK11* in HeyA8, OVCAR8, and FT190 cells has been described previously [26].

### 2.3. RNAi-Dependent Targeting of STK11 and STRADA

Transient *STK11* and *STRADA* knockdown in EOC cells was performed using DharmaFECT1 (Dharmacon, Thermo Fisher Scientific, Mississauga, ON, Canada), according to the manufacturer’s protocol. The final transfection reagent dilution was 1/500 for the 10 nM *STRADA* ON-TARGETplus SMARTpool (Dharmacon, L-005343-00-0005), *STK11* siGenome SMARTpool (Dharmacon, M-005035-02), and ON-TARGETplus non-targeting pool (siNT; Dharmacon, D-001810-10). The transfection reagent and siRNA were incubated for 20 min in 200 µL of serum-free media. The serum-rich medium was added to a final volume of 2 mL before cell incubation. After 24 h, 3 mL of serum-rich medium was added to the wells and incubated for 48 h. The cells were detached using 0.25% trypsin-EDTA (Gibco, Thermo Fisher Scientific, Mississauga, ON, Canada), counted, and seeded for further experiments. The downregulation of *STK11* and/or *STRADA* was verified by reverse transcription–quantitative PCR (qPCR) and immunoblotting.

### 2.4. Antibodies and Reagents

Primary antibodies were used against fibronectin (Abcam, Cambridge, UK, ab2413), LKB1 (CST, 3050), STRADα (Abcam, ab192879), vinculin (Sigma, V9264), and actin (Sigma, Markham, ON, Canada, A2066). Secondary horse radish peroxidase-conjugated antibodies against rabbit IgG (Cytiva, Vancouver, BC, Canada, NA934) were used. All antibodies were diluted in Tris-buffered saline-Tween 20 containing 5% bovine serum albumin. Cell viability was assessed using CellTiter-Glo (Promega, Madison, WI, USA) and alamarBlue (Thermo Fisher Scientific, DAL1100), while the Caspase-Glo 3/7 Assay (Promega) and CyQUANT Cell Proliferation Assay (Invitrogen, Thermo Fisher Scientific, Mississauga, ON, Canada, C35007) measured caspase 3/7 activity and DNA abundance, respectively. The reattached spheroids were fixed and stained with Hema3^TM^ (Thermo Fisher Scientific, 22-122911). Tissue culture-treated plates and Transwell membranes were coated with 20 μg/cm^2^ rat-tail collagen (Gibco, A10483-01), 20 μg/cm^2^ Matrigel (Corning, Fisher Scientific, Ottawa, ON, Canada, CLS356231), or 5 μg/cm^2^ fibronectin (Sigma, F0556).

### 2.5. Generating Whole-Cell Lysates

For spheroid whole-cell lysates, 5 × 10^5^ cells were seeded per well of a 6-well ULA plate. After 72 h, the spheroids were transferred to a conical tube and centrifuged in a swinging bucket rotor (1000× *g* at 4 °C for 3 min). The medium was aspirated, resuspended in 1 × cold PBS, and centrifuged again. Cell pellets were then lysed in modified RIPA buffer and clarified by centrifugation (15,000× *g* at 4 °C for 20 min), as previously described [27]. For adherent whole-cell lysates, the medium was aspirated, and the cells were washed with 1× cold PBS prior to lysis in modified RIPA buffer.

### 2.6. Polyacrylamide Gel Preparation and Immunoblot Analysis

Polyacrylamide gels were cast using 30% acrylamide/bis solution (37.5:1; Bio-Rad, 1610158). The gels were run at 120 volts for 90 min using a Bio-Rad Mini-PROTEAN II Electrophoresis System (Bio-Rad, Mississauga, ON, Canada). Proteins were transferred to PDVF membranes (Bio-Rad) at 100 volts for 70 min. PDVF membranes were blocked in Tris-buffered saline-Tween 20 containing 5% skim milk for 1 h. PDVF membranes were first incubated with primary antibodies overnight at 4 °C, and then with secondary antibodies for 1 h at room temperature. All immunoblots were exposed to an enhanced chemiluminescence substrate and analyzed using a ChemiDoc imaging system (Bio-Rad). Densitometry was performed using the Image Lab 6.0.1 software package (Bio-Rad).

### 2.7. RNA Isolation and Reverse Transcription–Quantitative PCR

Spheroids were lysed in 350 μL of RLT buffer and stored at −80 °C until processing. RNA was extracted and purified using the RNEasy Spin Column kit (Qiagen, Toronto, ON, Canada, 74104), according to the manufacturer’s protocol, with optional DNase I (Qiagen, 79254) treatment. RNA concentration and purity were assessed using a NanoDrop One Microvolume UV–Vis spectrophotometer (Thermo Fisher Scientific). cDNA was synthesized from 2 μg of purified RNA per reaction using the High-Capacity cDNA Reverse Transcription Kit (Thermo Fisher Scientific), according to the manufacturer’s protocol. cDNA was synthesized in a MyCycler thermocycler (Bio-Rad) programmed using the following protocol: 25 °C for 10 min, 37 °C for 120 min, and 85 °C for 5 min. qPCR master mixes were generated using Brilliant II SYBR Green QPCR Master Mix (Agilent Technologies, Mississauga, ON, Canada) according to the manufacturer’s protocol. Primer sequences were obtained from https://www.origene.com and were purchased from Invitrogen (Supplemental Table S1). The QuantStudio 3 RT-PCR System (Thermo Fisher Scientific), using built-in settings for SYBR Green Chemistry, cycled the reactions, which were analyzed using QuantStudio Design and Analysis Software 1.4.3. The 2^−ΔΔCT^ method was used to calculate the fold change difference relative to the siNT controls.

### 2.8. Transcriptome Analysis

The Human Clariom S microarray (Thermo Fisher Scientific) was used to assess the transcriptomes of wild-type and *STK11* KO OVCAR8 spheroids, the results from which were previously described [27].

### 2.9. Organoid Culture, Growth Analysis, and Lysis

EOC cells were resuspended as droplets using Cultrex Basement Membrane Extract (BME) PathClear Type 2 (R&D Systems, Toronto, ON, Canada) and cultured in Advanced DMEM/F-12 (Invitrogen) supplemented with Forskolin (Sigma), B-27™ (Invitrogen), Recombinant Human Noggin (R&D Systems), GlutaMAX™ (Invitrogen), N-Acetyl-L-cysteine (Sigma), Human EGF (Peprotech Inc., Cranbury, NJ, USA), HEPES (Wisent, Ste. Jean-Baptiste, Canada), Human FGF-10 (Peprotech Inc.), nicotinamide (Sigma), and ROCK inhibitor (Y27632 dihydrochloride, Sigma). Over 21 d of culture, an IncuCyte S3 System (Sartorius, Oakville, ON, Canada) imaged and quantified organoid number and area every 12 h using the Organoid Analysis Software. After 21 d of culture, the Cultrex BME matrix was dissolved in 500 uL of Cell Recovery Solution (Corning) and incubated on ice for 2 h. The organoids were collected and lysed in modified RIPA buffer, as described for generating whole-cell lysates.

### 2.10. Protein Extraction, Tandem Mass Tag (TMT) Labelling, and Mass Spectrometer Analysis

OVCAR8 wild-type and OVCAR8 *STK11*KO spheroids were lysed using a buffer containing 200 mM 4-(2-hydroxyethyl)-1-piperazinepropanesulphonic acid (EPPS; pH 8.6), 6 M guanidine, 1 mM PMSF, 100 mM NaF, and a phosphatase inhibitor cocktail (2 mM NaF, 2 mM imidazole, 1.15 mM Na_2_MoO_4_, 1 mM Na_4_P_2_O_7_, 4 mM Na_2_C_4_H_4_O_6_, 2 mM Na_3_VO_4_, and 1 mM β-glycerophosphate). Lysates were incubated in the dark with 5 mM Tris (2-carboxyethyl) phosphine solution and 15 mM indole-3-acetic acid for 30 and 45 min, respectively, and then quenched with 5 mM dithiothreitol. Sera-Mag^TM^ SpeedBeads (GE Healthcare, Little Chalfont, UK; 65152105050250) were added to the lysates, followed by equal volumes of 100% ethanol. The resulting mixture was incubated on a shaker for 10 min. The supernatant was removed from the mixture and the beads were washed and resuspended in 50 mM EPPS buffer (pH 8.5). After the beads and EPPS buffer were subjected to a 2 h LysC digestion at 1 mAu per 100 μg of protein, trypsin was added at a 1:50 ratio for overnight digestion. The beads were washed with 30% acetonitrile the following day to elute the peptides, which were stored at −80 °C.

Prior to TMT labelling, the peptides were resuspended in 50 mM EPPS and dried. The peptides were resuspended in 11-plex labelling TMT (Thermo Fisher Scientific, A34808) for 1 h. After confirming labelling efficiency, 2.7 μL of 5% hydroxylamine was added to each sample. The TMT-labelled samples were pooled and desalted using ZipTip (Millipore Sigma, ZTC18S096) to decrease the sample pH to <3. The TMT-labelled samples were loaded onto a C18 column, washed using 0.1% FA, and eluted with 70% ACN with 0.1% FA.

For mass spectrometry, the peptides were analyzed using a Q Exactive Plus mass spectrometer coupled with an EASYLCn-1000 system (Thermo Fisher Scientific). Peptides were loaded onto an Easy-LCn-1000 and separated on an EASY-Spray ES803A analytical column of 75 μm × 500 mm at 45 °C (Thermo Fisher Scientific) and a flow rate of 300 nl/min. Raw mass spectrometry data were processed using FragPipe (version 20.0) and Rstudio with the Tidyverse R package for data manipulation, the mice R package for imputing missing data, and the LIMMA R package for differential expression analysis.

### 2.11. Microscopy

Brightfield and mKate2/GFP fluorescence images of spheroids, reattached spheroids, Transwell membranes, scratch wound closure, and mesothelial clearance assays were captured using a DMI 4000B inverted microscope (Leica, Wetzlar, Germany) or the IncuCyte S3 Live-Cell Analysis System (Sartorius). The reattached spheroids were also imaged using a Zeiss AxioZoom V16 microscope (Zeiss, Toronto, ON, Canada).

### 2.12. Scratch Wound Closure Migration Assay

Confluent cell monolayers were scratched with a pipette tip and immediately imaged (0 h). For HeyA8, OVCAR8, and FT190 cell lines, images were acquired up to 24 h post-scratch, whereas OVCAR3 and OVCAR4 cell lines were imaged up to 48 h post-scratch. Each treatment was imaged six times at the same location for each time point post-scratch. ImageJ (version 2.0) was used to measure the scratch width, as the scratch diminished in cells migrating into the scratch area.

### 2.13. Transwell Migration and Invasion Assay

Transwell chambers with a pore size of 8 μm (Corning, Fisher Scientific, CLS3464) were used to assess cell or spheroid cell migration. For each EOC cell line, 25,000 cells or 5 spheroids were seeded into the top chamber in 200 μL of serum-free media, whereas serum-rich media were placed in the lower chamber. For spheroid experiments, 2000 EOC cells were seeded in 96-well ULA round-bottom plates (Corning) to form spheroids for 24 h. Transwell chambers coated with rat-tail collagen, Matrigel, and/or ZT-GFP mesothelial cells were used to assess cell invasion. Following invasion, the Transwell chambers were washed with 1× PBS and cotton swabs, fixed with 10% formalin (Thermo Fisher Scientific) for 10 min, and stained with DAPI for 10 min. The membranes were cut and mounted on microscope slides (Thermo Fisher Scientific, 12-550-17) using Immu-Mount (Invitrogen, P36980) and glass coverslips (Electron Microscopy Sciences, Hatfield PA, USA, 72230-01). Images were captured using a DMI 4000B inverted microscope, and cells that migrated from the upper to the lower chamber were counted using ImageJ.

### 2.14. Spheroid Reattachment Assay

Spheroids were formed by seeding 2000 EOC cells, or FT190 cells, in 96-well round-bottom ULA plates. Spheroids were grown for 72 h prior to being reattached to a standard cell-culture-treated 96-well plate. Images were captured using a DMI 4000B inverted microscope at 24 and 48 h post-reattachment. ImageJ was used to quantify the distance that cells migrated away from the reattached spheroids. Reattached spheroids were stained with Hema3 and imaged using a Zeiss AxioZoom V16 microscope.

### 2.15. Mesothelial Clearance Assay

Standard 24-well plates or the top chambers of Transwell membranes were coated with rat-tail collagen prior to seeding 100,000 or 10,000 GFP-labelled ZT mesothelial cells per well, respectively. EOC spheroids expressing nuclear mKate2 (red) were seeded on top of a confluent monolayer of mesothelial cells, where an IncuCyte S3 Live-Cell Analysis System imaged the spheroid and mesothelial cell monolayer every 4 h. After 24 h, a DMI 4000B inverted microscope was used to manually image the spheroids and mesothelial cells. ImageJ was used to quantify the area of mesothelial cells displaced by EOC spheroid cells, which were standardized to the initial EOC spheroid area.

### 2.16. Embedding Spheroids in Matrigel

Spheroids were generated by seeding 2000 EOC cells stably expressing nuclear-localized mKate2 (red) or GFP (green) in a 96-well round-bottom ULA plate. This experiment was also performed by seeding a 1:1 mixture of red or green wild-type and green or red *STK11* KO cells. The spheroids were grown for 24 h and incubated at 4 °C for 20 min before the addition of Matrigel. Spheroids embedded in 0, 2, 5, 10, 25%, or 50% Matrigel were placed at 4 °C for 20 min and centrifuged (200× *g* at 4 °C for 3 min). Images were acquired using an IncuCyte S3 Live-Cell Analysis System every 12 h for up to 96 h. Spheroid size was quantified using spheroid imaging analysis software on the IncuCyte S3 Live-Cell Analysis System, whereas ImageJ quantified the invasion area of EOC spheroid cells invading the surrounding Matrigel.

### 2.17. Zymography

ZT-GFP, HeyA8, HeyA8 *STK11*KO, OVCAR8, and OVCAR8 *STK11*KO cells, or spheroid cells, treated with either siNT or si*STRADA*, were serum-starved for 48 h. The conditioned media were concentrated using centrifugal filtering units (Millipore Sigma, Oakville, ON, Canada UFC801024) with pore sizes of 10 kDa, which were centrifuged in a swinging bucket rotor (2000× *g* at 4°C for 30 min). A 1:1 mixture of 2× sample preparation buffer lacking 2-mercaptoethanol was gently mixed with the concentrated conditioned media. The resulting conditioned media/sample preparation buffer mixture was not boiled or vortexed, and was loaded into a 1 mm polyacrylamide gel containing gelatin type A (BioShop, Burlington, ON, Canada, GEL771.100) and sucrose (BioShop, SUC700.1). After the gels were run at 100 volts for 2 h, the matrix matalloproteinases (MMPs) were renatured by washing the gels with renaturing buffer (1:40 Triton X-100 in deionized water) for 1 h at room temperature. Renatured MMPs were activated by incubating the gels with the developing buffer (Supplemental Table S2) at 37 °C for 16 h. The gels were stained with Coomassie Brilliant Blue G-250 (BioShop, CBB555) and diluted in acetic acid, methanol, and deionized water for 1 h. Excess Coomassie stain was removed from the gels by incubating them in a destaining solution containing acetic acid, methanol, and deionized water for 30 min. The gels were imaged using a ChemiDoc imaging system, and densitometry was performed using the Image Lab software package.

### 2.18. Statistical Analysis

Statistical significance was evaluated using Student’s *t*-test, or one-way or two-way ANOVA followed by Tukey’s multiple comparison tests. Statistical analyses were performed using GraphPad Prism Software 10.1 and *p*-values < 0.05 were considered statistically significant.

## 3. Results

### 3.1. LKB1 and STRADα Regulate EOC Cell Migration and Invasion

The mechanism by which AMPK-independent LKB1 signalling regulates EOC metastasis in mice remains unknown [26]. More clinically relevant, the role of LKB1 and its binding partner STRADα on human EOC invasion is also unknown. To this end, we first assessed the efficacy of *STK11* KO, *STK11* knockdown, and *STRADA* knockdown in EOC and FT190 cells (Supplemental Figure S1A) and EOC spheroids (Supplemental Figure S1B). We assessed *STRADA* knockdown efficiency 72 and 144 h post-transfection for EOC cell monolayers and spheroids, respectively. After verifying *STK11* KO and sufficient *STK11* or *STRADA* knockdown by RNA interference, we investigated the importance of *STK11* and *STRADA* in EOC cell migration using a basic scratch wound assay. Loss of LKB1 or STRADα significantly decreased scratch closure in HeyA8 cells but not in the other EOC cell lines tested (Supplemental Figure S2A). We repeated the scratch wound assay with the non-cancerous FT190 cell line and found that the downregulation of LKB1 or STRADα disrupted cell motility too (Supplemental Figure S2B).

We next investigated if the loss of LKB1 and/or STRADα impacted cell viability or proliferation to determine whether the migration differences observed are due to intrinsic migratory behavior or differences in proliferation/viability. The loss of LKB1 decreased the viability of HeyA8, OVCAR8, and FT190 cells, whereas the loss of STRADα decreased the viability of OVCAR4 and FT190 cells (Supplemental Figure S3A). Although caspase 3/7 activity remained unchanged in EOC cells and FT1190 cells (Supplemental Figure S3B), downregulating LKB1 significantly decreased DNA abundance in HeyA8, OVCAR8, and FT190 cells, suggesting that LKB1 regulates cell proliferation in these cell lines (Supplemental Figure S3C). Given that alterations in cell proliferation may confound the scratch wound assay results, we next assessed EOC cell migration and invasion using Transwell chamber assays coated with rat-tail collagen or Matrigel as surrogate basement membranes. The downregulation of LKB1 and STRADα decreased HeyA8 cell invasion across the Matrigel and collagen-coated Transwell membranes, but significantly increased OVCAR8, OVCAR3, and OVCAR4 cell invasion (Supplemental Figure S4A). We repeated the experiment with FT190 cells, and as observed in the scratch assay, the downregulation of LKB1 and STRADα reduced invasion (Supplemental Figure S4B). Our results suggest that LKB1 and STRADα are essential for fallopian tube cell migration and invasion; however, in EOC, the outcomes may be cell-line-specific.

### 3.2. Reduced LKB1 and STRADα Alter EOC Spheroid Growth and Viability

**Figure 1:**
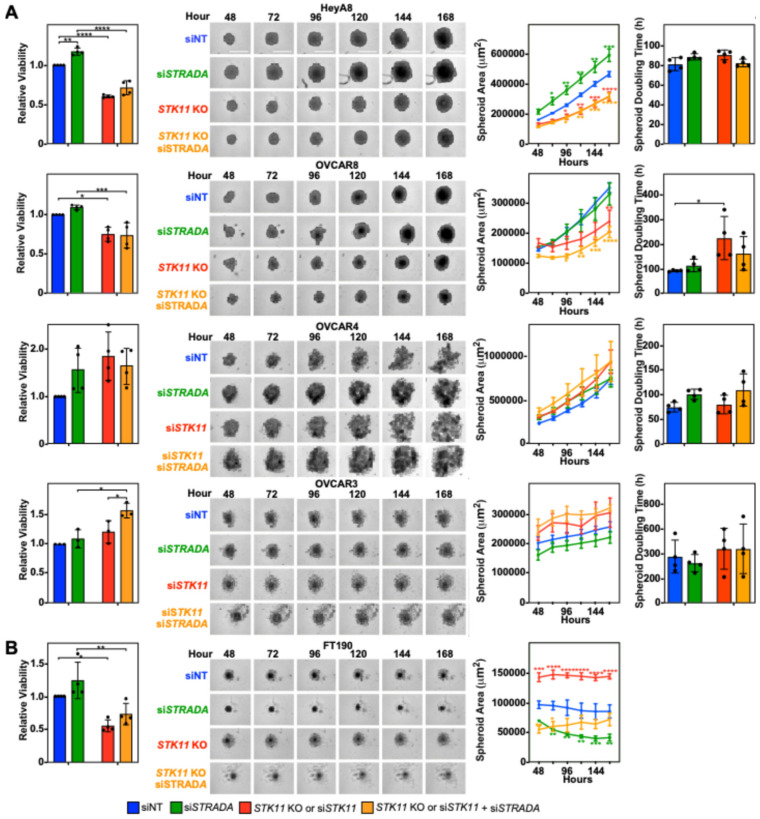
LKB1 and STRADα regulate EOC spheroid size and viability. (**A**) HeyA8, HeyA8 *STK11* KO, OVCAR8, and OVCAR8 *STK11* KO cells were transfected with 10 nM siNT or 10 nM si*STRADA* for 72 h. OVCAR3 and OVCAR4 cells were transfected with 10 nM siNT, 10 nM si*STRADA*, or 10 nM si*STK11* for 72 h. (**B**) FT190 and FT190 *STK11* KO cells were transfected with 10 nM siNT or 10 nM si*STRADA* for 72 h. For each experimental condition, 2000 cells were seeded in a 96-well ULA cluster plate and imaged every 24 h using an IncuCyte S3 Live-Cell Analysis System. Representative brightfield images of each experimental condition were captured 48–168 h post-seeding for all cell lines. CellTiter-Glo assessed spheroid cell viability at the 168 h time point, and representative graphs for relative viability are found to the left of the representative brightfield images. Spheroid area was measured using the Sartorius spheroid module analysis tool, and spheroid doubling time was calculated using the rate of change of the spheroid area. Representative graphs for spheroid area and spheroid doubling time are located to the right of the representative brightfield images. The quantification represents three to five independent experiments (mean ± SD). Significance is indicated as * = *p* < 0.05, ** = *p* < 0.01, *** = *p* < 0.001, and **** *p* < 0.0001. Scale bars = 0.5 mm.

Given the variability observed between EOC cell lines, we next investigated the roles of LKB1 and STRADα on EOC spheroid migration and invasion, as spheroids more accurately recapitulate EOC pathobiology. We first examined the impact of downregulating LKB1 and STRADα on spheroid cell growth and viability, processes which are essential for spheroid-mediated metastasis [28]. We observed that the loss of LKB1 decreased HeyA8 and OVCAR8 spheroid area and viability, whereas STRADα knockdown increased HeyA8 spheroid area and viability. Although no differences were observed in OVCAR3 and OVCAR4 spheroid areas, the downregulation of both proteins increased OVCAR3 spheroid viability (Figure 1A). Since LKB1 reduced the proliferation of cells cultured in monolayer (Supplemental Figure S3C), we next measured spheroid doubling time and observed a significant increase in OVCAR8 spheroid doubling time (Figure 1A). Therefore, in well-established EOC cell lines, it appears that spheroid viability is linked to LKB1 and/or STRADα activity in a diverse manner, with some lines showing a clear loss of growth and others being unaffected. As a noncancerous cell line, we did not expect FT190 cells to form robust spheroids. Indeed, the few FT190 spheroids that formed did not grow in suspension, leading to a discrepancy between the spheroid area and viability (Figure 1B). For this reason, spheroid doubling time could not be quantified and the use of FT190 cells in subsequent spheroid experiments was not pursued.

### 3.3. Decreased LKB1 and STRADα Antagonize EOC Mesothelial Clearance

**Figure 2:**
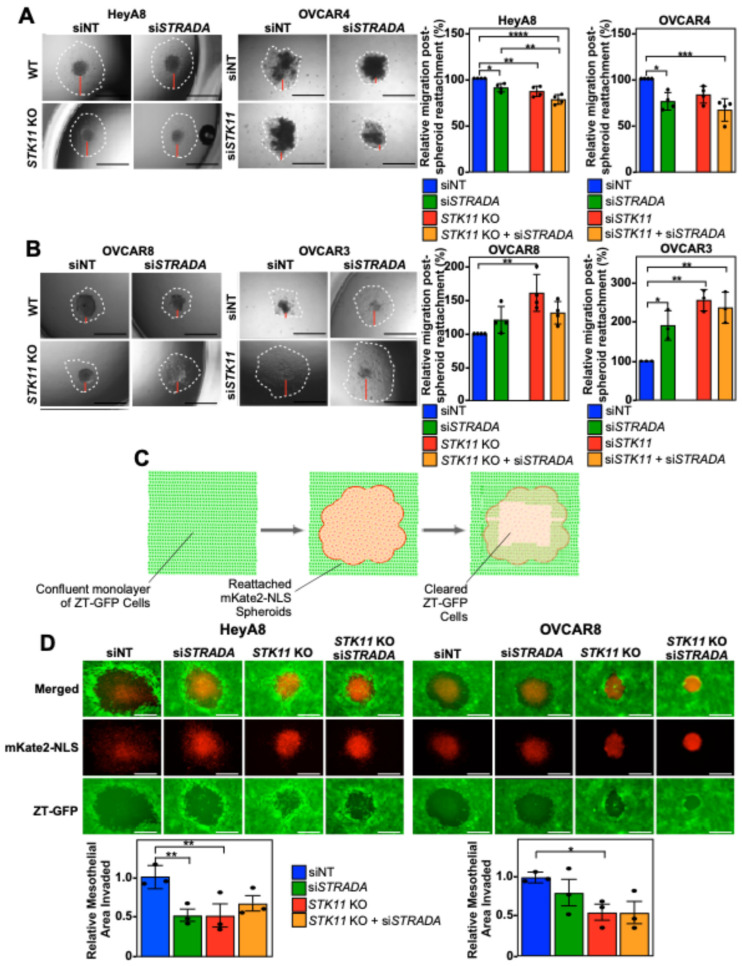
EOC spheroid cell dispersion and mesothelial clearance are dependent on LKB1 and STRADα. HeyA8, HeyA8 *STK11* KO, OVCAR8, and OVCAR8 *STK11* KO cells were transfected with 10 nM siNT or 10 nM si*STRADA* for 72 h. OVCAR3 and OVCAR4 cells were transfected with 10 nM siNT, 10 nM si*STRADA*, or 10 nM si*STK11* for 72 h. For all treatments, 2000 cells were seeded in 96-well ULA plates to form spheroids for 24 h. Spheroids were reattached using standard tissue-culture-treated 96-well plates and imaged after 24 h (HeyA8 and OVCAR8) or 48 h (OVCAR3 and OVCAR4) using a Leica DMI 4000B inverted microscope. ImageJ was used to quantify initial spheroid size and cell migration post-reattachment relative to siNT. Graphs from three independent experiments (mean ± SD) are located to the right of the representative images. Based on each cell line’s response to the downregulation of *STK11* or *STRADA*, (**A**) reattached HeyA8 and OVCAR4 spheroids were paired and (**B**) reattached OVCAR8 and OVCAR3 spheroids were paired. Black scale bars = 1 mm and red bars represent one of four quadrants measured to quantify relative migration post-spheroid reattachment. (**C**) A schematic representation of mesothelial clearance assay. ZT-GFP human mesothelial cells (in green) are displaced by re-attaching EOC spheroids (in red) expressing mKate2 conjugated to a nuclear localization signal (NLS). (**D**) HeyA8, HeyA8 *STK11* KO, OVCAR8, and OVCAR8 *STK11* KO cells stably expressing mKate2-NLS (red) were transfected with 10 nM siNT or 10 nM si*STRADA* for 72 h. For all experimental conditions, 2000 cells were seeded in 96-well ULA plates and grown for 24 h. EOC spheroids were reattached to a confluent monolayer of ZT-GFP mesothelial cells cultured on collagen-coated 24-well plates. Images of mesothelial clearance at 24 h post-reattachment were captured using a Leica DMI 4000B inverted microscope. ImageJ was used to quantify EOC spheroid area and mesothelial clearance area. Relative mesothelial clearance for each treatment was standardized to original spheroid size. Scale bars = 0.5 mm. Significance is indicated as * = *p* < 0.05, ** = *p* < 0.01, *** = *p* < 0.001, and **** = *p* < 0.0001.

We next investigated whether human EOC spheroid reattachment, an essential step in the spheroid-mediated metastatic process, relies on the LKB1-STRADα pathway. The downregulation of LKB1 and STRADα decreased cell dispersion in the reattached HeyA8 and OVCAR4 spheroids (Figure 2A). Alternatively, downregulating LKB1 and STRADα protein levels increased the cell dispersion of reattached OVCAR8 and OVCAR3 spheroids (Figure 2B). Therefore, it appears that spheroid reattachment is linked to LKB1 and/or STRADα activity in a diverse manner, with some lines showing a clear reduction or increase in reattachment and dispersion on tissue-culture-treated plates.

Since the invasion of the mesothelium is paramount for efficient EOC metastasis throughout the peritoneal cavity [29], we next investigated whether the mesothelial clearance of EOC is regulated by LKB1 and STRADα. As OVCAR3 and OVCAR4 cells formed loosely packed spheroids of variable sizes with longer cell dispersion times (Figure 1 and Figure 2), we limited the mesothelial clearance assay to HeyA8 and OVCAR8 spheroids. Interestingly, after reattaching EOC spheroids (red) to a confluent monolayer of ZT-GFP mesothelial cells (green; Figure 2C), we found that downregulating LKB1 significantly decreased mesothelial clearance in both cell lines; however, STRADα knockdown only decreased HeyA8 spheroid mesothelial clearance (Figure 2D).

### 3.4. Reduced LKB1 and STRADα Disrupt EOC Spheroid Cell Invasion Through In Vitro Models of the Mesothelial Lining

As mesothelial clearance precedes peritoneal organ invasion, we examined the roles of LKB1 and STRADα in EOC spheroid cell migration and invasion. For Transwell chamber experiments, OVCAR3 and OVCAR4 spheroids were omitted because, compared to HeyA8 and OVCAR8 spheroids, they formed loosely packed spheroids that did not efficiently migrate or invade across the membranes (Supplemental Figure S5). Continuing our investigation with HeyA8 and OVCAR8 spheroids, we observed that the downregulation of LKB1 and STRADα decreased the number of cells migrating through the membrane (Supplemental Figure S6A). In support of these findings, Transwell membranes coated with rat-tail collagen showed that *STK11* KO and/or *STRADA* knockdown significantly decreased the number of invading HeyA8 and OVCAR8 spheroid cells (Supplemental Figure S6B).

**Figure 3:**
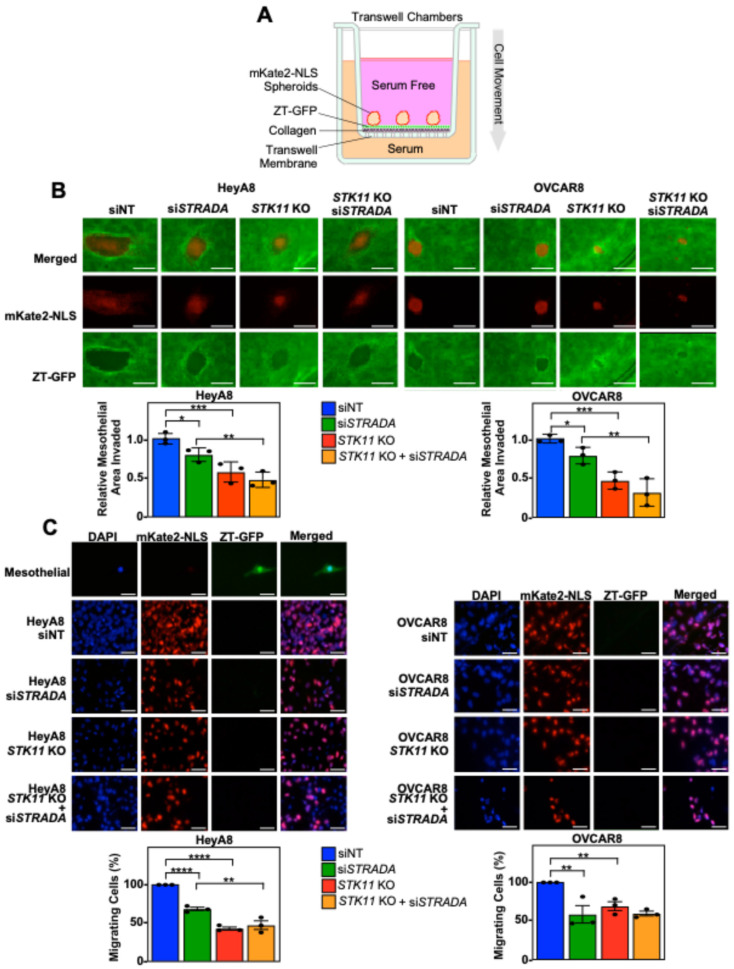
Downregulating LKB1 and STRADα disrupts invasion, as established through 3D in vitro models of mesothelial layers. (**A**) A schematic representation of the mesothelial invasion assay. In the Transwell chamber, the upper chamber was filled with serum-free medium and the lower chamber was filled with serum-rich medium, separated by a porous Transwell membrane. The Transwell membranes were coated with rat-tail collagen followed by a confluent monolayer of ZT-GFP mesothelial cells (green). mKate2-NLS-expressing EOC spheroid cells (red) were reattached to the mesothelial layer: EOC spheroid cells cleared the mesothelial layer, reorganized the underlying collagen, and invaded across the membrane to the lower chamber. (**B**) HeyA8, HeyA8 *STK11* KO, OVCAR8, and OVCAR8 *STK11* KO cells stably expressing mKate2-NLS (red) were transfected with 10 nM siNT or 10 nM si*STRADA* for 72 h. For all experimental conditions, 2000 EOC cells were seeded in 96-well ULA plates and grown for 24 h. EOC spheroids were reattached to a confluent monolayer of ZT-GFP mesothelial cells cultured on rat-tail collagen in the top chamber of a Transwell membrane. A Leica DMI 4000B inverted microscope was used to image mesothelial clearance 24 h post-reattachment. ImageJ was used to quantify EOC spheroid area and mesothelial clearance area. The mesothelial clearance for each treatment was standardized to the original spheroid size and siNT was set to 1. Scale bars = 0.5 mm. (**C**) Spheroid cell invasion was assessed 48 h post-seeding. The Transwell membranes were fixed, stained with DAPI, and mounted on microscope slides. Images of DAPI (blue), mKate2-NLS (red), and GFP (green) were acquired using a Leica DMI 4000B inverted microscope, and DAPI-stained nuclei overlapping with mKate2-NLS were counted using ImageJ. The percentage of invading cells for each experimental condition was standardized to the siNT control. Scale bars = 40 μm. The quantification represents three independent experiments (mean ± SD). Significance is indicated as * = *p* < 0.05, ** = *p* < 0.01, *** = *p* < 0.001, and **** = *p* < 0.0001.

Next, we modeled EOC invasion across the peritoneal lining by reattaching mKate2-NLS-expressing EOC spheroids to a confluent monolayer of ZT-GFP cells on a Transwell membrane coated with rat-tail collagen. Prior to reaching the bottom Transwell chamber, spheroid cells cleared the mesothelial layer and invaded through the pores of a collagen-coated Transwell membrane towards the serum-containing media (Figure 3A). We observed that both *STK11* KO and *STRADA* knockdown decreased mesothelial clearance in both cell lines (Figure 3B). Few ZT-GFP mesothelial cells invaded the Transwell membrane, whereas the number of HeyA8 and OVCAR8 cells was significantly decreased by downregulating LKB1 and STRADα (Figure 3C). Therefore, LKB1-STRADα signalling pathways are essential for EOC spheroids to disperse the mesothelial lining and invade underlying matrix proteins.

### 3.5. LKB1 and STRADα Loss Decrease EOC Spheroid Invasion Through Matrigel

Knowing that LKB1 enhanced migration and invasion through the mesothelial layer and across Transwell membranes, we next recapitulated in vivo conditions using Matrigel to model the ECM proteins present in ovarian tumors and malignant ascites. We observed that lower concentrations of Matrigel (2–25%) substantially increased HeyA8 and OVCAR8 spheroid sizes under all experimental conditions but did not stimulate EOC spheroid invasion into the surrounding matrix. Alternatively, EOC spheroid cells can be tracked by invading away from the parent spheroids embedded in high Matrigel concentrations (50%). For HeyA8 spheroids, the downregulation of LKB1 and/or STRADα had no impact on the total nuclear mKate2 area of spheroids embedded in 2–25% Matrigel, whereas LKB1 knockout significantly reduced the nuclear mKate2 area of spheroids embedded in 50% Matrigel. For OVCAR8 spheroids, we found that downregulating *STK11* significantly decreased the nuclear mKate2 area under all experimental conditions (Supplemental Figure S7).

**Figure 4:**
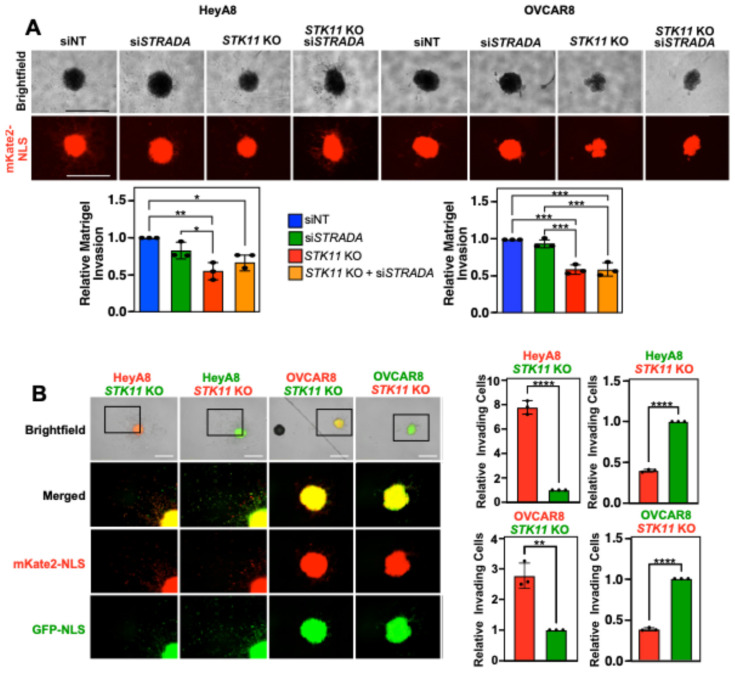
Downregulating LKB1 and STRADα disrupts EOC spheroid invasion through Matrigel. (**A**) HeyA8, HeyA8 *STK11* KO, OVCAR8, and OVCAR8 *STK11* KO cell lines expressing mKate2-NLS (red) were transfected with 10 nM siNT or 10 nM si*STRADA* for 72 h. After seeding 2000 cells per well in a 96-well ULA plate for 24 h, spheroids were embedded in Matrigel at a final concentration of 50%. After 72 h, spheroid invasion area was quantified using ImageJ and normalized to the siNT controls from three independent experiments. Graphs of relative Matrigel invasion are located below representative brightfield and mKate2-NLS images. (**B**) HeyA8 or OVCAR8 wild-type and *STK11* KO cell lines expressing nuclear-localized mKate2 (red) or GFP (green) were mixed in a 1:1 ratio. For each cell line, 1000 wild-type and 1000 *STK11* KO cells were seeded per well in a 96-well ULA plate to generate mixed spheroids. After 24 h, spheroids were embedded in a final concentration of 50% Matrigel. An IncuCyte S3 Live-Cell Analysis System imaged the spheroids 72 h post-Matrigel embedding, and ImageJ was used to quantify the number of invading cells. Graphs from three independent experiments (mean ± SD) are shown and significance is indicated as * = *p* < 0.05, ** = *p* < 0.01, *** = *p* < 0.001, and **** = *p* < 0.0001. Scale bars = 1 mm.

Given that invasion was detected when spheroids were embedded in the highest concentration of Matrigel (50%), we more closely assessed the relative extent of Matrigel invasion by removing the 0–25% Matrigel conditions, performing fewer pairwise analyses, and analysing the corresponding brightfield images. Although the degrees of significance may have changed, the overall conclusions remain that *STK11* KO decreased spheroid cell Matrigel invasion in both cell lines while *STRADA* knockdown showed no clear loss of EOC spheroid cell Matrigel invasion (Figure 4A). Next, we seeded spheroids with both wild-type and *STK11* KO cells to assess whether the loss of invasion observed in *STK11* KO cells could be rescued by the proteins secreted by wild-type cells or through wild-type-*STK11* KO cell interactions. Each cell line expressed nuclear mKate2 (red) and GFP (green). Regardless of which nuclear fluorescent signal was expressed by *STK11* KO cells, there were significantly fewer invading cells, even when isogenic cells expressing LKB1 were present within spheroids (Figure 4B). As a result, we provide evidence that LKB1 activity is important for EOC spheroid invasion through a matrix, and co-culturing both wild-type and *STK11* KO cells does not salvage the loss of invasion observed in *STK11* KO cells.

### 3.6. LKB1 Ablation in EOC Spheroid Cells Decreases MMP Activity

**Figure 5:**
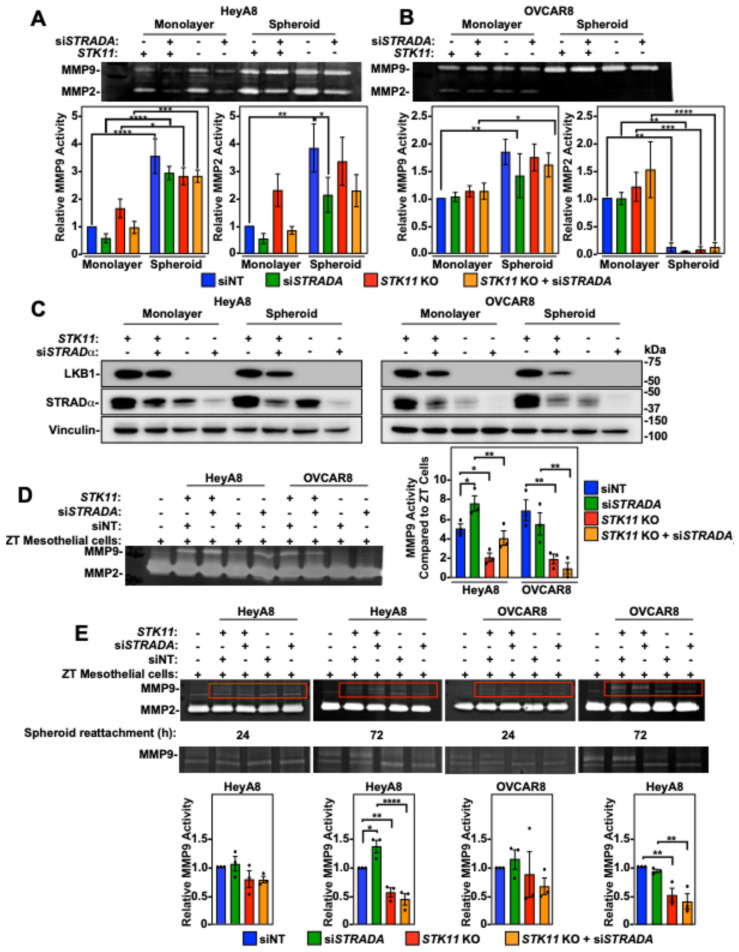
The impact of LKB1 and STRADα on matrix metalloproteinase (MMP) 2/9 activity. HeyA8, HeyA8 *STK11* KO (**A**), OVCAR8, and OVCAR8 *STK11* KO (**B**) cells were transfected with 10 nM siNT or 10 nM si*STRADA* for 72 h. All experimental conditions were reseeded in a standard 6-well tissue-culture-treated plate (monolayer condition) or in a 96-well ULA plate (spheroid condition). Both monolayer cells and spheroids were serum-starved for 48 h, and the conditioned media were concentrated prior to zymographic assays. Gels for zymography contained gelatin and were incubated in MMP-reactivating buffers for 16 h. After MMP reactivation, the gels were stained with Coomassie Brilliant Blue G-250 prior to imaging and densitometry. Quantification represents relative MMP9 and MMP2 activities relative to the siNT control in monolayer cells, as determined from three independent experiments (mean ± SD). (**C**) HeyA8 and OVCAR8 cell monolayers and reattached spheroids responsible for generating the conditioned media utilized in zymography were lysed and subjected to SDS-PAGE and immunoblotting using antibodies specific for LKB1, STRADα, and vinculin. (**D**) HeyA8, HeyA8 *STK11* KO, OVCAR8, and OVCAR8 *STK11* KO cells stably expressing mKate2-NLS (red) were transfected with 10 nM siNT or 10 nM si*STRADA* for 72 h. For all experimental conditions, 2000 cells were seeded in 96-well ULA plates and grown for 24 h. The EOC spheroids were reattached to a confluent monolayer of ZT-GFP mesothelial cells cultured on rat-tail collagen. After 72 h of mesothelial clearance, EOC spheroids and ZT cell co-cultures were serum-starved for 48 h to generate conditioned media for zymographic assays. Quantifications of MMP9 activity relative to ZT-GFP mesothelial cells from three independent experiments (mean ± SD) are graphed to the right of the representative zymographic blots. (**E**) EOC spheroids were reattached to a confluent monolayer of ZT-GFP cells 24 or 72 h prior to zymography. Red boxes in upper zymograms are re-displayed below for better visualization of MMP9 activity. Quantifications of MMP9 activity relative to siNT controls from three independent experiments (mean ± SD) are graphed below representative zymographic blots. Significance is indicated as * = *p* < 0.05, ** = *p* < 0.01, *** = *p* < 0.001, and **** = *p* < 0.0001.

Since matrix metalloproteinase 9 (MMP9) and MMP2 are upregulated in ovarian cancer and their activity is correlated with invasion and metastasis [30,31], we hypothesized that the impact of LKB1 and STRADα on EOC invasion is mediated by MMP activity. We utilized zymographic assays to measure MMP2 and MMP9 activity and found increased levels in HeyA8 spheroid cultures compared to monolayer cells. Furthermore, *STRADA* knockdown decreased MMP9 and MMP2 activity in HeyA8 cell monolayers and spheroid cultures. Alternatively, LKB1 ablation increased MMP activity in HeyA8 monolayer cells, but had no impact on spheroids (Figure 5A). In the OVCAR8 cell line, spheroid culture significantly increased MMP9 activity but decreased MMP2 activity, whereas the downregulation of *STK11* or *STRADA* had no effect on MMP activity (Figure 5B). Cell monolayers and spheroids were lysed and subjected to SDS-PAGE and immunoblotting to verify LKB1 and STRADα downregulation (Figure 5C).

Based upon our results described above, the impact of LKB1 and STRADα on EOC invasion could not be explained by MMP activity when the EOC spheroids were cultured alone. Therefore, we investigated the MMP activity of EOC spheroids reattached to a confluent monolayer of ZT mesothelial cells. In the absence of EOC spheroids, ZT cells showed low MMP9 activity and robust MMP2 activity. MMP2 activity was unchanged by the reattachment of EOC spheroids, whereas MMP9 activity was significantly increased. Specifically, reattaching EOC spheroids with STRADα knockdown demonstrated a significant increase in MMP9 activity under HeyA8 experimental conditions. However, reattaching HeyA8 and OVCAR8 spheroids with ablated LKB1 levels showed reduced MMP9 activity compared to that in wild-type EOC spheroids (Figure 5D). Therefore, LKB1 inactivation decreases MMP9 activity in the presence of a mesothelial monolayer.

Due to the challenge of distinguishing MMP activity between mesothelial cells and EOC spheroid cells, we reattached EOC spheroids and allowed short-term (24 h) or prolonged (72 h) mesothelial cell dispersion. As the duration of EOC spheroid cell dispersion or mesothelial clearance increased, the *STK11* KO-dependent loss of MMP9 activity became statistically significant (Figure 5E). Therefore, EOC spheroids and the mesothelium must be in contact for extended durations to upregulate MMP9 activity, which is dependent on intact LKB1 signalling.

### 3.7. Downregulation of LKB1 and STRADα Decreased Spheroid Invasion Through Loss of Fibronectin

**Figure 6:**
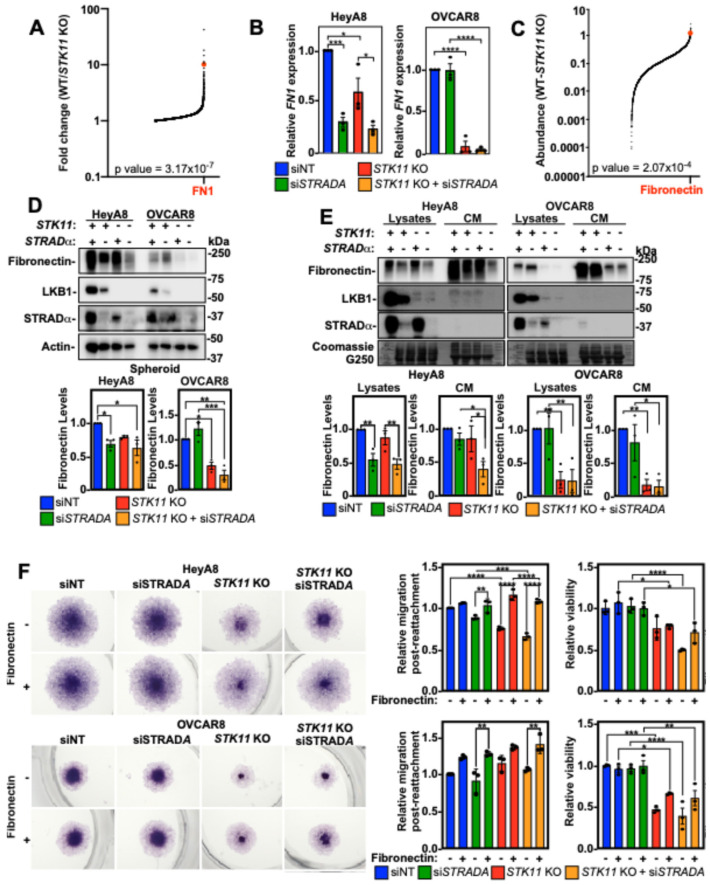
Fibronectin restores *STK11* KO cell migration post-spheroid reattachment. (**A**) Global transcriptome analysis of OVCAR8 wild-type (WT) and OVCAR8 *STK11* KO cultured as 24 h spheroids using the Affymetrix Human Clariom S microarray. The fold changes of mRNA transcripts expressed in OVCAR8/OVCAR8 *STK11* KO spheroids were graphed, and fibronectin (*FN1*) mRNA expression is indicated (red dot). Only mRNAs with a greater expression in OVCAR8 spheroids are represented in the graph. (**B**) RT-qPCR was performed to assess *FN1* mRNA expression in HeyA8, HeyA8 *STK11* KO, OVCAR8, and OVCAR8 *STK11* KO spheroids transfected with 10 nM siNT or 10 nM si*STRADA* for 72 h. *FN1* expression was normalized to siNT controls from three independent experiments (mean ± SD). (**C**) Proteomic analysis of OVCAR8 wild-type (WT) and OVCAR8 *STK11* KO spheroids grown for 24 h. Spheroids were lysed and subjected to tandem mass tag isobaric labelling and mass spectrometry analysis. All detectable peptides of the kinome were graphed as OVCAR8-OVCAR8 *STK11* KO, and fibronectin protein abundance is indicated (red dot). Only proteins with a greater abundance in OVCAR8 cells are represented in the graph. (**D**) HeyA8, HeyA8 *STK11* KO, OVCAR8, and OVCAR8 *STK11* KO cells were transfected with 10 nM siNT or 10 nM si*STRADA* for 72 h. Cells were reseeded into 6-well ULA plates to generate spheroids, and after 72 h, spheroids were lysed for SDS-PAGE and immunoblotting using antibodies specific for fibronectin, LKB1, STRADα, and actin. LKB1 and STRADα verified knockout and knockdown, respectively, whereas total fibronectin was quantified, standardized to actin, and graphed below representative immunoblots relative to siNT control from three independent experiments (mean ± SD). (**E**) HeyA8, HeyA8 *STK11* KO, OVCAR8, and OVCAR8 *STK11* KO cells were transfected with 10 nM siNT or 10 nM si*STRADA* for 72 h. The conditioned media (CM) were isolated and prepared for immunoblotting and the cells that produced the CM were lysed and subjected to SDS-PAGE and immunoblotting using antibodies specific for fibronectin, LKB1, and STRADα. The blots were stripped and stained with Coomassie Brilliant Blue G250, which functioned as a loading control. Total fibronectin was quantified for cell lysates and CM, standardized to Coomassie Brilliant Blue G250, and graphed. The graphs are shown below representative immunoblots relative to siNT control from three independent experiments (mean ± SD). (**F**) HeyA8, HeyA8 *STK11* KO, OVCAR8, and OVCAR8 *STK11* KO cells were transfected with 10 nM siNT or 10 nM si*STRADA* for 72 h. Spheroids grown for 24 h in 96-well ULA plates were reattached for 48 h to standard tissue-culture-treated plates or 5 µg/cm^2^ fibronectin-coated plates. Cell viability was assessed by an alamarBlue assay followed by fixation and cell staining using Hema3. The relative migration and cell viability was quantified, and graphs from three independent experiments (mean ± SD) are shown to the right of the representative micrographs. Significance is indicated as * = *p* < 0.05, ** = *p* < 0.01, *** = *p* < 0.001, and **** = *p* < 0.0001.

Although LKB1 and STRADα in EOC spheroids regulate MMP9 activity in mesothelial cells, this does not account for our results of LKB1 and STRADα loss in the invasion observed in experiments without ZT cells. Given the importance of ECM components in spheroid formation and invasion [32], we assessed the influence of decreasing LKB1 and STRADα protein levels on ECM components. Using an Affymetrix Human Clariom S microarray on OVCAR8 wild-type and *STK11* KO spheroids, we found that fibronectin (*FN1*) transcript levels were significantly higher in wild-type spheroids than in *STK11* KO spheroids (Figure 6A). In support of these results, we observed via RT-qPCR that the downregulation of LKB1 or STRADα reduced *FN1* expression in EOC spheroids (Figure 6B). The proteomic analysis of OVCAR8 wild-type and *STK11* KO spheroids identified fibronectin as one of the most differentially expressed proteins (Figure 6C). Immunoblotting the cell lysates of HeyA8 and OVCAR8 spheroids verified that LKB1 ablation and STRADα knockdown reduced fibronectin protein abundance (Figure 6D). We next assessed the relative amount of fibronectin secreted by HeyA8 and OVCAR8 spheroid cells and observed that when LKB1 or STRADα were reduced, there was a significant decrease in secreted fibronectin (Figure 6E).

Given that decreasing LKB1 and STRADα expression reduced fibronectin protein abundance, we coated tissue culture plates with fibronectin and reattached spheroids to assess cell dispersion/migration. Fibronectin coating increased cell migration in si*STRADA* and *STK11* KO HeyA8 cells with no impact on viability. In fact, there were no significant differences between siNT and *STK11* KO/si*STRADA* experimental conditions when the plate was coated with fibronectin, suggesting that fibronectin is essential for HeyA8 spheroid cell migration upon reattachment. Although LKB1 and STRADα loss had little impact on OVCAR8 spheroid cell migration, we observed that fibronectin increased migration post-reattachment under all experimental conditions. However, this was only statistically significant for the *STRADA* knockdown. Although fibronectin did not affect OVCAR8 spheroid cell viability, the loss of viability observed due to LKB1 loss in these cells was partially rescued (Figure 6F). Therefore, the loss of LKB1 activity downregulates *FN1*, which dampens EOC invasion independently of MMP expression or activity.

### 3.8. LKB1 Inactivation Disrupts EOC Organoid Growth

**Figure 7:**
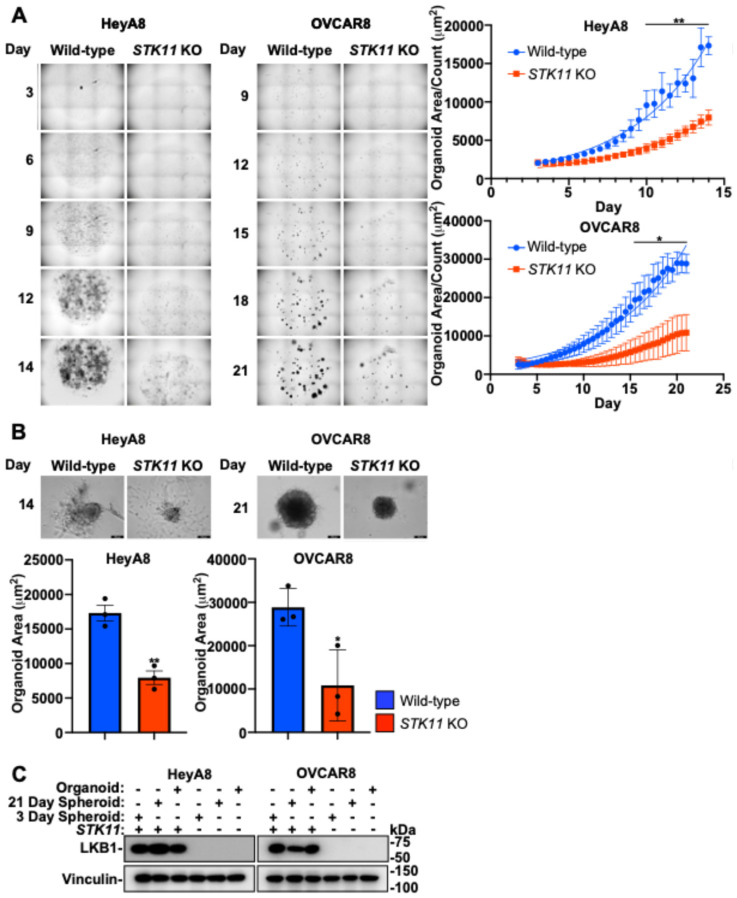
LKB1 inactivation disrupts EOC organoid growth. (**A**) HeyA8, HeyA8 *STK11* KO, OVCAR8, and OVCAR8 *STK11* KO cell lines were seeded in Cultrex BME with modified organoid growth medium and imaged with an IncuCyte S3 Live-Cell Analysis System every 12 h for 14 days (HeyA8) or 21 days (OVCAR8). The IncuCytes organoid analysis software calculated total organoid area (µm^2^) and organoid count per image. Graphs of average organoid size (i.e., organoid area/count) are located to the right of the representative micrographs. (**B**) Brightfield images of the EOC organoids were acquired using a Leica DMI 4000B inverted microscope after 14 days (HeyA8) or 21 days (OVCAR8) of growth. Graphs from three independent experiments (mean ± SD) are located below the representative images and significance is indicated as * = *p* < 0.05 and ** = *p* < 0.01. Scale bars = 200 μm. (**C**) HeyA8 and OVCAR8 organoids and spheroids cultured for 3 or 21 days were lysed and subjected to SDS-PAGE and immunoblotting using antibodies specific for LKB1 and vinculin.

The disruption of the LKB1 pathway through *STK11* KO impedes EOC spheroid formation, while downregulating LKB1 or STRADα decreases cell migration, mesothelial clearance, and invasion. We have shown previously in xenografted mice that LKB1 may facilitate the growth and establishment of secondary metastases [26]. To further assess LKB1-STRADα in this final step of advanced EOC pathogenesis, we utilized an in vitro matrix-bound organoid model system to assess metastasis formation. After 14 days (HeyA8) or 21 days (OVCAR8) in culture, LKB1 inactivation decreased the average organoid size, which was exacerbated over time (Figure 7A). Upon termination of the experiment, we assessed the size of individual organoids and verified that LKB1 inactivation decreased the organoid size. As observed in Matrigel-embedded spheroids (Figure 4), *STK11* KO reduced cell invasion from these Cultrex-embedded organoids, suggesting that this phenotype is reproducible in different matrices and 3D model systems of EOC (Figure 7B). Following analysis, organoids were lysed and subjected to SDS-PAGE and immunoblotting alongside 3- and 21-day spheroids to verify *STK11* KO (Figure 7C). Therefore, LKB1 may be important for the establishment of metastatic niches, as organoid growth and matrix invasion are enhanced by intact LKB1 signalling.

## 4. Discussion

**Figure 8:**
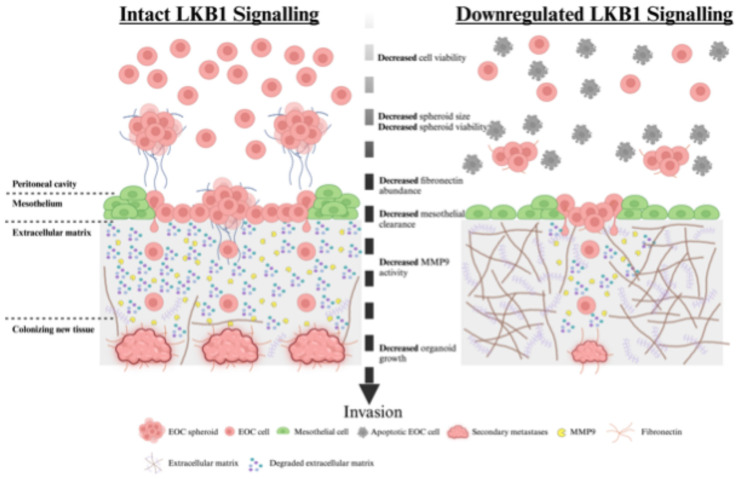
Proposed model of LKB1 pathway activity during ovarian cancer metastasis. EOC cells disseminate from the primary ovarian tumor and form multicellular spheroids in the peritoneal cavity. EOC spheroids colonize distant sites by invading across the mesothelial lining of the peritoneal organs, reorganizing the underlying extracellular matrix, and establishing secondary tumors. Our in vitro models of EOC metastasis indicated that intact LKB1 signalling is important for EOC spheroid formation and viability in suspension, mesothelial clearance after reattachment, extracellular matrix remodeling, and new secondary tumor growth. The ablation of LKB1 expression decreased each of these key metastatic steps, which may be due to reduced viability, fibronectin production, or MMP9 activity.

Historically, LKB1 has commonly been described as having tumor suppressor properties [22]. Evidence exists for its potential tumor suppressor role in EOC, including blocking epithelial-to-mesenchymal transition and apoptosis resistance in EOC cells [33], and LKB1 loss can induce papillary serous carcinoma when combined with other genetic mutations [34]. However, there is mounting evidence that LKB1 activity is required in advanced-stage cancers. LKB1 is considered an upstream master regulator of metabolism that controls the altered utilization of lipids, carbohydrates, and proteins during times of nutrient deprivation by activating catabolic processes, such as autophagy [35,36,37]. We have recently shown that LKB1 is essential for EOC spheroid viability [14], as it activates reactive oxygen species scavenging through NFκB upregulation, which further supports EOC spheroid cell survival [38]. In fact, our previous report using spheroid and mouse xenograft models of metastatic EOC suggested that intact LKB1 is required for disease dissemination. Here, we provide new evidence that downregulating the LKB1 pathway decreases mesothelial clearance, invasion, and organoid growth in relevant in vitro models of EOC metastasis (Figure 8). Despite differences in cell proliferation and spheroid viability outcomes, the migration differences observed are likely due to a combination of alterations in intrinsic migratory behavior, as well as proliferation/viability. Nevertheless, LKB1 pathway inactivation by either *STK11* genetic ablation or *STRADA* knockdown decreased EOC spheroid mesothelial clearance, invasion through matrices, and metastatic colony growth. Significantly, we utilized EOC organoids as a three-dimensional model for secondary metastases and observed that LKB1 downregulation may be sufficient to delay metastases formation. Given that spheroids and organoids recreate the pathophysiology of EOC, our data suggest that LKB1 inactivation slows metastatic disease.

Given that siRNA is a transient method of gene knockdown, the persistence of siRNA-mediated knockdown is crucial when interpreting its impact on key processes like cell migration and invasion. If the knockdown is short-lived, cells may regain normal levels of the targeted protein over time, potentially affecting the observed phenotypic changes. Here, we address *STK11* and *STRADA* knockdown efficiencies in Supplemental Figure S1. Since the monolayer and spheroid lysates were generated 72 h and 144 h post-transfection, respectively, the only results in this manuscript where the transient knockdown extends beyond these time points are the spheroid growth analyses in Figure 1. Regardless, the statistical difference in spheroid size was maintained after the 72 and 96 h time points, suggesting that the siRNAs were not losing their effect.

Intact LKB1 signalling in EOC cells and spheroids is essential for efficient metastasis in mice [26]. In support of this, we demonstrated that LKB1 and its direct binding partner, STRADα, promote EOC mesothelial cell clearance, spheroid invasion, and organoid growth. Our investigation of these properties uncovered two independent mechanisms that rely on LKB1-STRADα signalling to facilitate EOC spheroid cell invasion. Initially, we observed a reduction in EOC spheroid invasiveness due to targeting LKB1 and STRADα which appeared to be independent of MMP2 and MMP9 activities native to EOC spheroid cells. However, when EOC spheroids were reattached to a mesothelial cell monolayer, MMP9 activity was downregulated by LKB1 and STRAD loss. Currently, it is unclear whether the altered MMP9 expression arises from EOC spheroid cells or from mesothelial cells as an interactive response in this co-culture system. MMP activity may be regulated by gene expression, pro-enzyme activation and inhibition, and enzyme localization [39]. Thus, future work will investigate how LKB1 activity may directly or indirectly affect MMP9 expression and activity in the tissue microenvironment of EOC metastasis.

The second process where we observed that intact LKB1 and STRADα expressions were required for EOC spheroid cell invasion was fibronectin production, which is known to serve as an ECM scaffold for invading tumor cells [40]. We observed that *FN1* expression and protein abundance were significantly impaired by *STK11* deletion or *STRADA* knockdown in the EOC spheroids. Furthermore, re-establishing a fibronectin substratum rescued the spheroid cell dispersion capacity when *STK11* or *STRADA* expression was lost. Previously, we observed that *FN1* expression is upregulated during spheroid formation and that genetic ablation of the LKB1 substrate *NUAK1* significantly abrogates fibronectin expression, thereby impacting spheroid formation [27]. In summary, our findings regarding LKB1-STRAD function in EOC spheroid cell invasion provide new mechanistic evidence to align with our previous report of the decreased metastatic capacity of EOC cells due to LKB1 loss in xenografted mice [26].

Although MMP activity and fibronectin abundance may account for some of the invasion observations, there must be additional intrinsic differences between wild-type and *STK11* KO spheroid cells. Reduced *STK11* KO spheroid cell invasion remains, despite being co-seeded with LKB1 intact cells, suggesting that MMP activity and fibronectin from the co-seeded cells cannot rescue the *STK11* KO spheroid cell invasion phenotype. Therefore, future work will aim to further distinguish spheroid cell autonomous *STK11* KO deficiencies that impact invasion potential.

The viscoelasticity and surface tension of the ECM can have a profound impact on the growth, proliferation, and migration of three-dimensional spheroids and organoids. Lower concentrations of the ECM have previously been reported to approximate the properties of ascites and support cell translocation [41], while elevated ECM concentrations have been used as in vitro metastasis models [42]. Here, we used fibronectin, collagen, Matrigel, and BME at varying concentrations to model the ECM found in the tumor microenvironment, malignant ascites, and sites for secondary metastases. Previous reports assessing ovarian cancer spheroids embedded in the ECM have found that at low ECM levels, EOC spheroids form outgrowths that are highly invasive. However, as the concentration of the ECM increases, outgrowths diminish and spheroid migration becomes restricted [41]. In contrast, we observed larger, loosely packed EOC spheroids when embedded in low concentrations of Matrigel; however, at higher Matrigel concentrations, spheroids were smaller, and this condition induced cell invasion. Likewise, we observed organoid cell growth and invasion throughout the high-concentration BME matrix, suggesting that EOC cell migration is not necessarily restricted by a stiffer ECM. EOC spheroid cell invasion was clearly diminished by the loss of LKB1 and STRAD. Importantly, this effect appeared to be intrinsic to cells lacking LKB1-STRAD activity, since only those cells with intact LKB1 could invade Matrigel from spheroids using mixed cell cultures. However, reattaching spheroids to fibronectin- and collagen-coated plates restored the cell dispersion capacity of the si*STRADA* or *STK11* KO spheroids.

We observed cell-line-dependent differences in the general invasive potential of the EOC spheroids. HeyA8 and OVCAR8 cells formed tight, rapidly growing spheroids, whereas OVCAR3 and OVCAR4 cells formed loosely packed spheroids. Indeed, HeyA8 and OVCAR8 cells demonstrated an invasive phenotype that was able to disperse and migrate away from spheroids or through Transwell membranes, whereas OVCAR3 and OVCAR4 cells were much less likely to invade. There are inherent differences among these cell lines, which may account for pathobiological discrepancies. OVCAR8 cells are categorized as high-grade serous adenocarcinoma, which is *a TP53*-mutated line, as are OVCAR3 and OVCAR4 cells, whereas HeyA8 cells are categorized as low-grade serous adenocarcinoma with no mutations in *TP53* [43]. These two EOC histotypes differ in their cell of origin for precursor lesions, degree of genomic instability, response to chemotherapy, and overall patient survival [43]. Despite these inherent differences, both HeyA8 and OVCAR8 cell lines demonstrated the requirements for LKB1 pathway regulation of spheroid cell viability, mesothelial clearance, invasion capacity, and the regulation of both MMP activity and fibronectin production.

The LKB1 enzyme complex functions as a heterotrimer in a 1:1:1 ratio of LKB1 protein to STRAD and Mouse protein 25 (MO25). The pseudokinase domain of STRAD binds to the LKB1 kinase domain, whereas MO25 binds to the C-terminal domain of STRAD. Together, the LKB1-STRAD-MO25 complex activates AMPK and several AMPK-related kinases to modulate metabolism, proliferation, polarity, migration, and energy expenditure. STRADα and STRADβ isoforms exist in mammals and share several functional similarities [44]. However, STRADα activated LKB1 more efficiently than STRADβ in vitro [45], and only *STRADA* KO could reduce LKB1 protein levels in the cerebral cortex of mice [44]. Therefore, we focused solely on the STRADα isoform. However, we appreciate the fact that some EOC cell lines may also rely on STRADβ for LKB1 complex activity. For instance, HeyA8 cells exhibited greater effects in response to STRADα knockdown than OVCAR8 cells. In contrast to HeyA8 cells, *STRADB* transcript levels were greater than those of *STRADA* in OVCAR8 cells; therefore, it is possible that LKB1 also utilizes STRADβ in OVCAR8 cells and spheroids for its invasive properties. Therefore, future studies could distinguish the functional differences or compensation between these STRAD proteins in their contribution to aberrant LKB1 activity in EOC.

It is currently understood that STRAD plays a major role in LKB1 regulation, but there is little known regarding other LKB1-independent STRAD activities. One group identified STRADα as a regulator of *Caenorhabditis elegans* cell polarity [46], and another found that STRAD participates in cancer cell polarity and migration in LKB1-null cells [20]. Throughout this investigation, we observed that the loss of STRAD induced significant changes that were not observed in *STK11* KO treatment. For instance, the HeyA8 spheroid area, viability, and MMP9 activity in the presence of ZT mesothelial cells were increased only by STRADα knockdown. As such, we propose that LKB1-independent STRAD activities may exist in EOC, which should be further explored in this context and expanded to broader cancer biology. If STRAD possesses LKB1-independent functions, solely blocking LKB1 expression may free up the other activities of STRAD, with unknown consequences on tumor pathogenesis.

Defining LKB1 as a tumor suppressor has hindered the development of inhibitory compounds targeting LKB1 as potential cancer therapeutics. Indeed, there are no known pharmacological agents that directly inhibit LKB1 activity. However, several studies have demonstrated that abrogating LKB1 improves the efficacy of other therapeutics. For example, repressing LKB1 activity with miR-17–92 sensitizes lymphomas to biguanide treatment [47]. Similarly, LKB1 knockdown sensitized endometrial cancer cells to metformin-mediated apoptosis [48]. In the proportion of non-small cell lung cancer patients that have STK11-inactivating mutations, this LKB1 deficiency increases cytotoxicity of ERK inhibitors [49]. Therefore, future investigations should seek to design novel inhibitors targeting LKB1 complex activity as a strategy to block stress-induced survival pathways in the context of advanced cancers like EOC, perhaps in combination with other therapeutics.

## Data Availability

Research data supporting this publication will be made publicly available upon publication.

## Supplementary Materials

The following supporting information can be downloaded at: https://www.mdpi.com/article/10.3390/cancers16223726/s1, Figure S1: Determining STK11 and STRADα knockdown and knockout efficiencies; Figure S2: Downregulating LKB1 and STRADα alters scratch wound closure of EOC cells and fallopian tube epithelial cells; Figure S3: Downregulating LKB1 and STRADα decreases proliferation in EOC cells and fallopian tube epithelial cells; Figure S4: LKB1 and STRADα regulate invasion of EOC cells and fallopian tube epithelial cells through Matrigel and collagen-coated Transwell membranes; Figure S5: Evaluation of EOC spheroid cell migration and invasion across Transwell membranes; Figure S6: Downregulating LKB1 and STRADα decreases EOC spheroid cell migration and invasion across Transwell membranes; Figure S7: Impact of LKB1 and STRADα loss on EOC invasion through Matrigel is dependent on matrix concentration. Table S1: Primer sets used for qPCR; Table S2: Developing buffer protocol.
