# Supplementary material for: LKB1 and STRADα Promote Epithelial Ovarian Cancer Spheroid Cell Invasion
Source: Cancers (Basel). 2024 Nov 5;16(22):3726. doi: 10.3390/cancers16223726 (PMC11591840; doi:10.3390/cancers16223726)
Supplement: Supplementary file 1 [file cancers-16-03726-s001.zip › cancers-3225867-supplementary/cancers-3225867-Supplemental Figures.pdf]

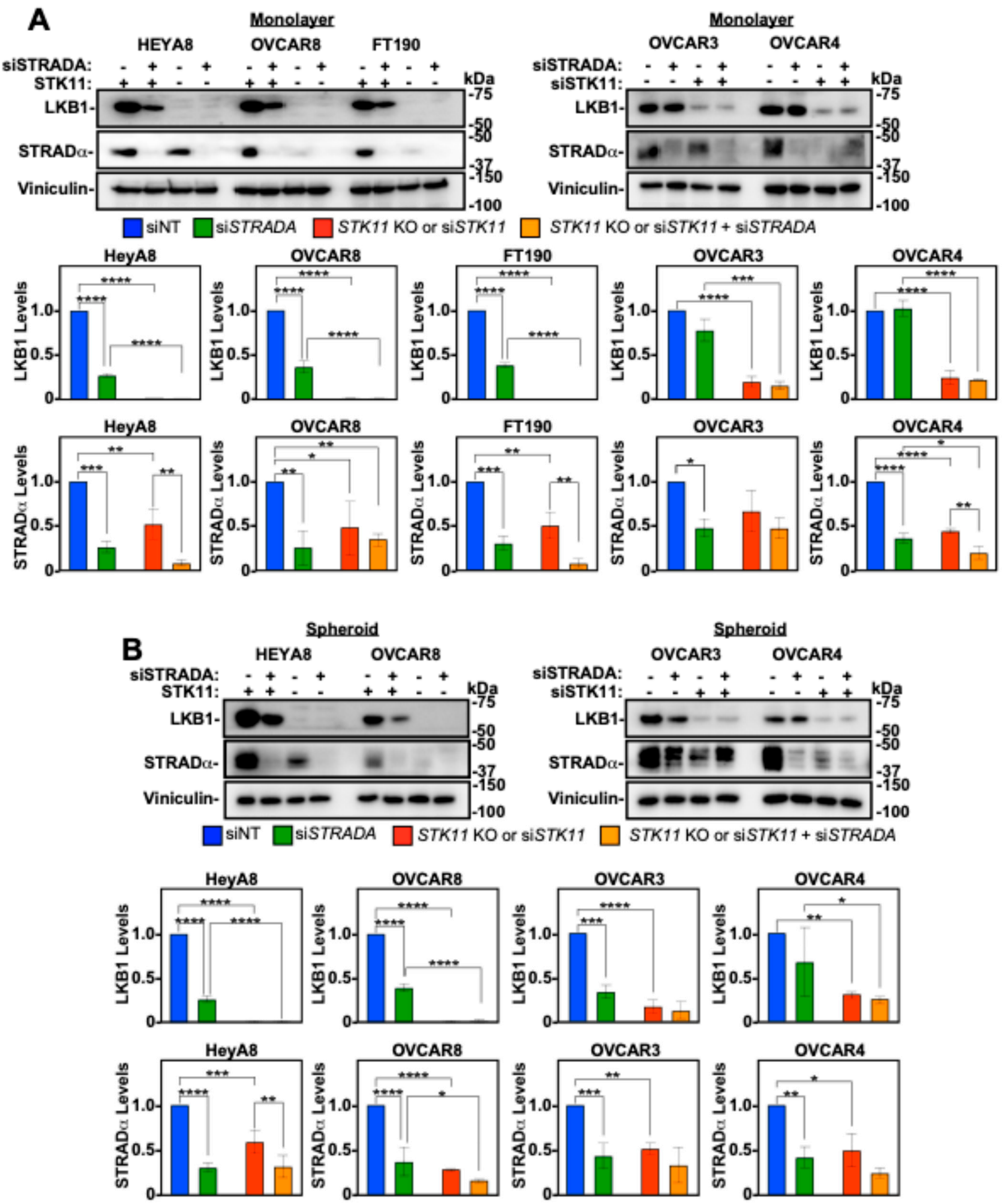

Supplemental Figure S1: Determining *STK11* and STRADα knockdown and knockout efficiencies.

(A) HeyA8, OVCAR8, FT190, OVCAR3, and OVCAR4 cells in monolayer were lysed and subjected to SDS-PAGE and immunoblotting using antibodies specific for LKB1, STRADα, and vinculin. LKB1 and STRADα were quantified, standardized to vinculin, and graphed below the representative immunoblots. Quantification was relative to the siNT control from three independent experiments (mean ± SD). (B) This experiment was repeated using HeyA8, OVCAR8, OVCAR3, and OVCAR4 spheroids. Significance is indicated as * = $P<0.05$, ** = $P<0.01$, *** = $P<0.001$, and **** = $P<0.0001$.

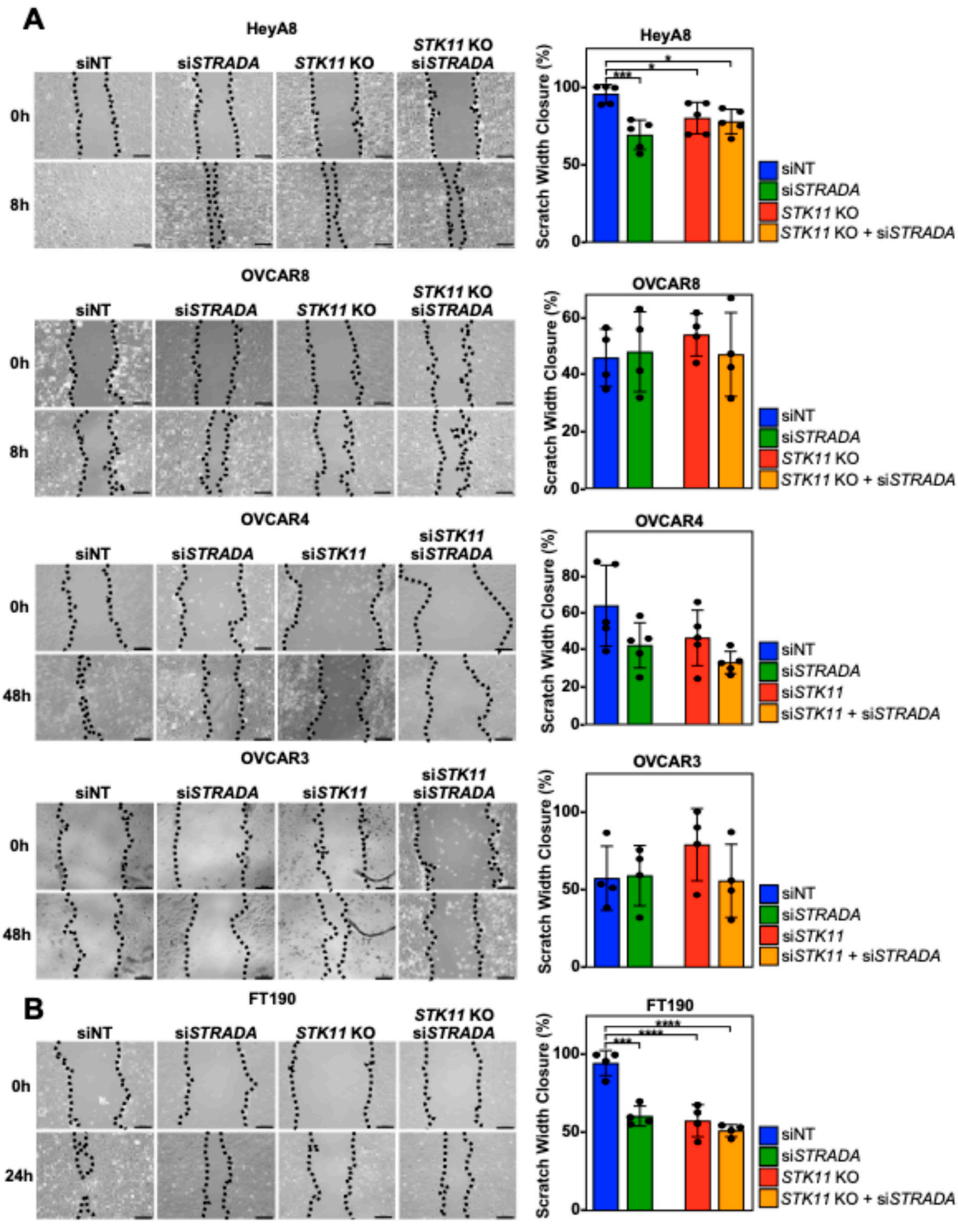
A
HeyA8
siNT
siSTRADA
STK11 KO
STK11 KO siSTRADA
0h
8h
Scratch Width Closure (%)
siNT
siSTRADA
STK11 KO
STK11 KO + siSTRADA
OVCAR8
OVCAR4
siSTK11
siSTK11 siSTRADA
48h
siSTK11 + siSTRADA
OVCAR3
B
FT190
24h

Supplemental Figure S2: Downregulating LKB1 and STRADα alters scratch wound closure of EOC cells and fallopian tube epithelial cells.

(A) HeyA8, HeyA8 *STK11* KO, OVCAR8, and OVCAR8 *STK11* KO cells were transfected with 10 nM siNT or 10 nM si*STRADA* for 72 h. OVCAR3 and OVCAR4 cells were transfected with 10 nM siNT, 10 nM si*STRADA* or 10 nM si*STK11* for 72 h. (B) FT190 and FT190 *STK11* KO cells were transfected with 10 nM siNT or 10 nM si*STRADA* for 72 h. A scratch was made in the confluent cell monolayer, and images were acquired immediately after the scratch (0 h) and 8, 24 or 48 h post-scratch using a Leica DMI 4000 B inverted microscope. The percent scratch width closure was quantified using ImageJ software, and data generated from four to five independent experiments (mean ± SD) were graphed to the right of representative micrographs. Significance is indicated as * = P<0.05, *** = P<0.001, and **** = P<0.0001. Scale bars = 200 µm.

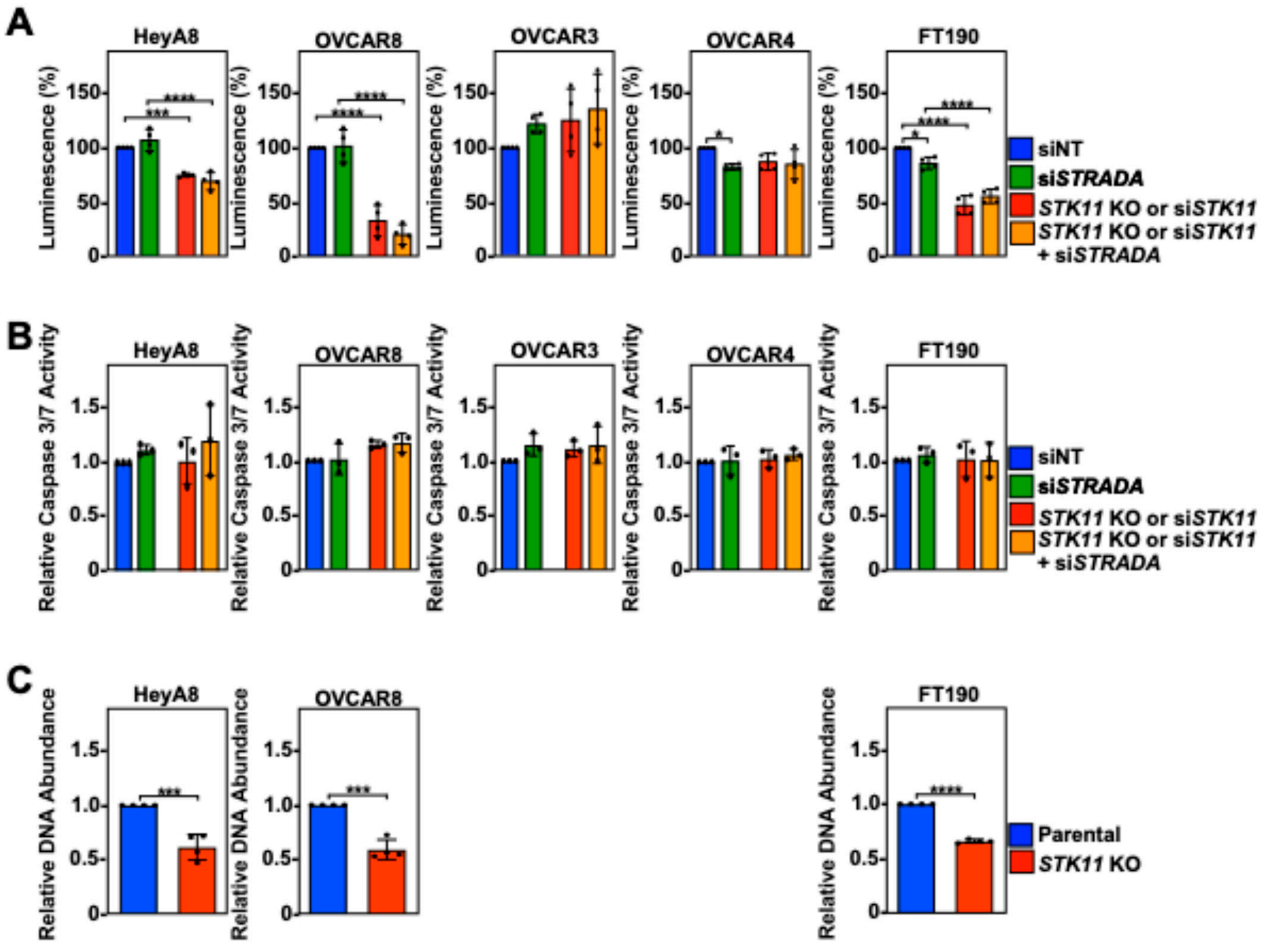

Supplemental Figure S3: Downregulating LKB1 and STRADα decreases proliferation in EOC cells and fallopian tube epithelial cells

HeyA8, HeyA8 *STK11* KO, OVCAR8, OVCAR8 *STK11* KO, FT190, and FT190 *STK11* KO cells were transfected with 10 nM siNT or 10 nM si*STRADA* for 72 h. OVCAR3 and OVCAR4 cells were transfected with 10 nM siNT, 10 nM si*STRADA* or 10 nM si*STK11* for 72 h. The CellTiter-Glo Luminescent viability

assay (A) and Caspase-Glo 3/7 assay system (B) assessed relative viability and Caspase 3/7 activity, respectively. CellTiter-Glo Luminescence (%) and relative Caspase 3/7 activity from three independent experiments (mean ± SD) were graphed. HeyA8, HeyA8 *STK11* KO, OVCAR8, OVCAR8 *STK11* KO, FT190, and FT190 *STK11* KO cells were lysed using the Cyquant cell proliferation assay and relative DNA abundance from three independent experiments (mean ± SD) were graphed (C). Significance is indicated as * = P<0.05, *** = P<0.001, and **** = P<0.0001.

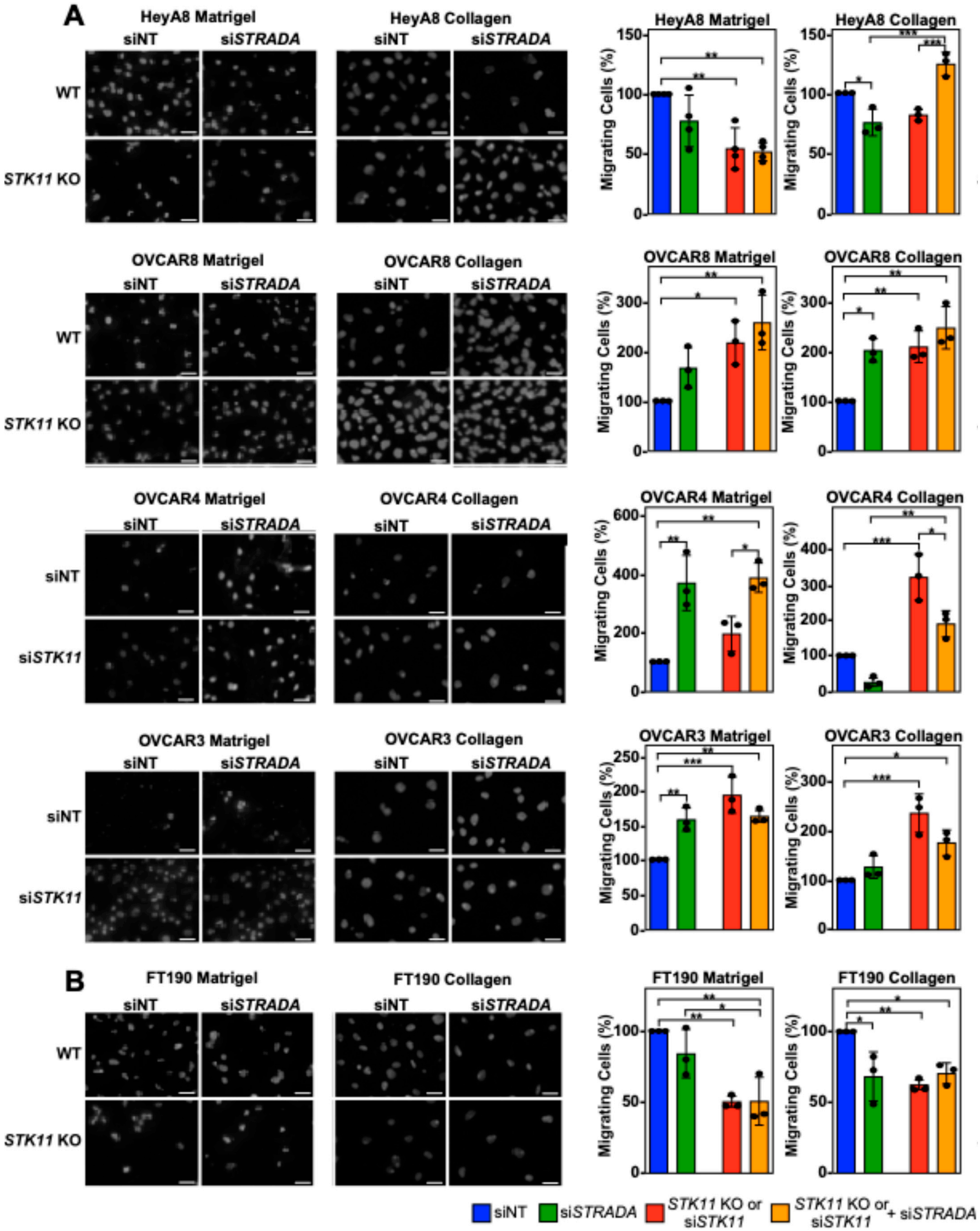
A
HeyA8 Matrigel
HeyA8 Collagen
siNT
siSTRADA
WT
STK11 KO
Migrating Cells (%)
OVCAR8 Matrigel
OVCAR8 Collagen
OVCAR4 Matrigel
OVCAR4 Collagen
siSTK11
OVCAR3 Matrigel
OVCAR3 Collagen
B
FT190 Matrigel
FT190 Collagen
siNT
siSTRADA
STK11 KO or siSTK11
STK11 KO or siSTK11 + siSTRADA

Supplemental Figure S4: LKB1 and STRADα regulate invasion of EOC cells and fallopian tube epithelial cells through Matrigel and collagen-coated Transwell membranes.
(A) HeyA8, HeyA8 *STK11* KO, OVCAR8, and OVCAR8 *STK11* KO cells were transfected with 10 nM siNT or 10 nM si*STRADA* for 72 h. OVCAR3 and OVCAR4 cells were transfected with 10 nM siNT, 10 nM si*STRADA* or 10 nM si*STK11* for 72 h. (B) FT190 and FT190 *STK11* KO cells were treated with 10 nM siNT or 10 nM si*STRADA* for 72 h. Transwell membranes were coated with Matrigel or rat-tail collagen and incubated at room temperature for 24 h prior to cell addition. For each experimental condition, 25,000 cells were transferred into Transwell inserts with serum-free medium in the upper chamber and high-serum (10% FBS) medium in the bottom chamber. Cell invasion was assessed 24 h (HeyA8, OVCAR8, and FT190) or 48 h (OVCAR3 and OVCAR4) post-seeding in which Transwell membranes were fixed, stained with DAPI, and mounted on microscope slides. Images were acquired using a Leica DMI 4000 B inverted microscope, and nuclei were counted using ImageJ. The percentages of migrating and invading cells for each experimental condition were standardized to that of the siNT control. Quantification represents three to four independent experiments (mean ± SD). Significance is indicated as * = P<0.05, ** = P<0.01, and *** = P<0.001. Scale bars = 40 μm.

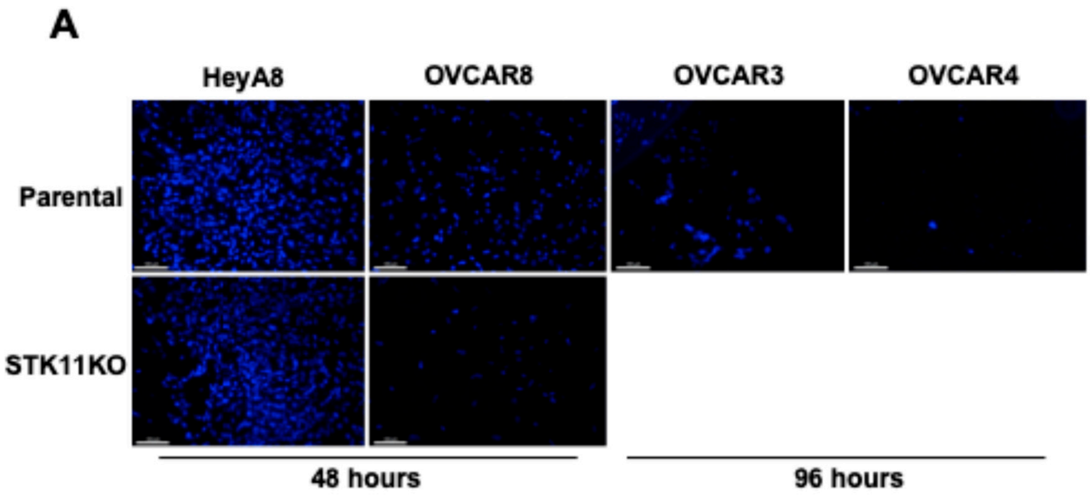

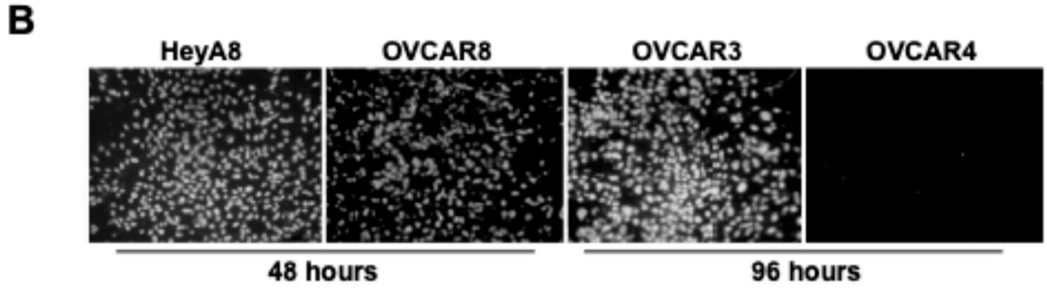

Supplemental Figure S5: Evaluation of EOC spheroid cell migration and invasion across Transwell membranes.

(A) HeyA8, HeyA8 *STK11* KO, OVCAR8, OVCAR8 *STK11* KO, OVCAR3, and OVCAR4 cells were seeded in a 96-well ULA plate at a density of 2,000 cells per well for 24 h. Spheroids were then transferred into Transwell inserts containing serum-free medium in the upper chamber, and high-serum (10% FBS) medium was placed in the bottom chamber. (B) Transwell membranes were coated with rat-tail collagen and incubated at room temperature for 24 h prior to spheroid addition. Spheroid cell migration and invasion were assessed 48- or 96 h post-seeding as indicated, in which Transwell membranes were fixed, stained with DAPI, and mounted on microscope slides. All images were acquired using a Leica DMI 4000B inverted microscope. Scale bars = 100 µm.

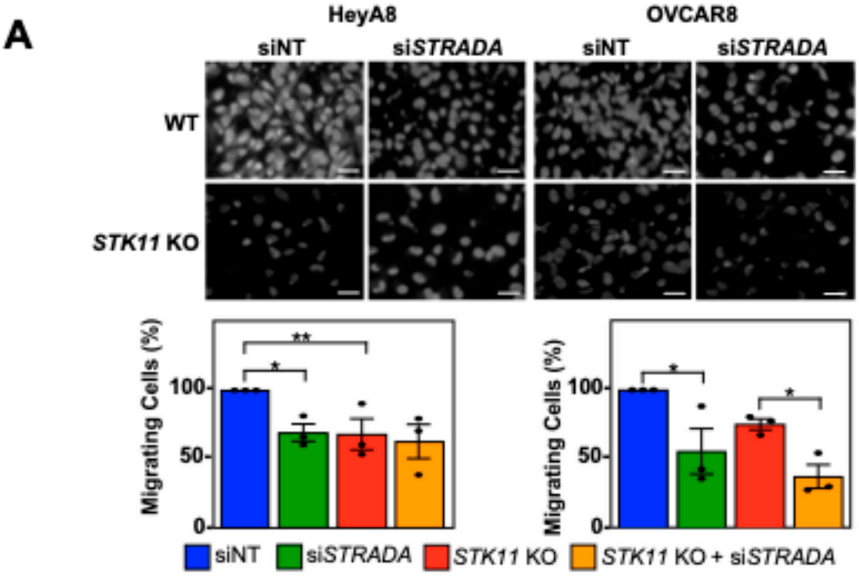

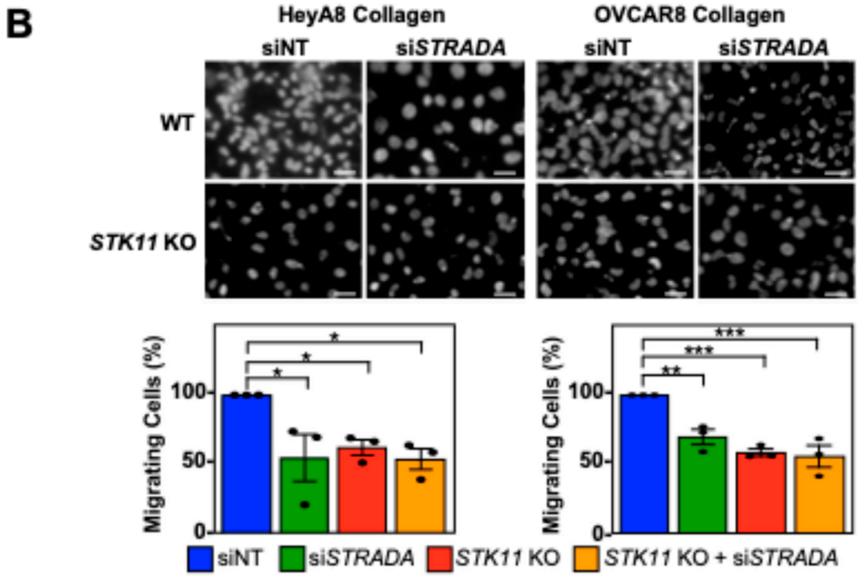

Supplemental Figure S6: Downregulating LKB1 and STRADα decreases EOC spheroid cell migration and invasion across Transwell membranes.

(A) HeyA8, HeyA8 *STK11* KO, OVCAR8, and OVCAR8 *STK11* KO cells were transfected with 10 nM siNT or 10 nM si*STRADA* for 72 h. Cells were seeded at 2000 cells per well in a 96-well ULA plate for 24 h to form spheroids. Five individual spheroids were transferred into Transwell inserts containing serum-free medium in the upper chamber, and high-serum (10% FBS) medium was placed in the bottom chamber. (B) Transwell membranes were coated with rat-tail collagen and incubated at room temperature for 24 h prior to spheroid addition. Spheroid cell migration and invasion were assessed 48 h post-seeding, in which Transwell membranes were fixed, stained with DAPI, and mounted on microscope slides. Images were acquired using a Leica DMI 4000B inverted microscope, and nuclei were counted using ImageJ. The percentages of migrating and invading cells under each experimental condition were standardized to the siNT control. The quantification represents three independent experiments (mean ± SD). Significance is indicated as * = $P<0.05$, ** = $P<0.01$, and *** = $P<0.001$. Scale bars = 40 μm.

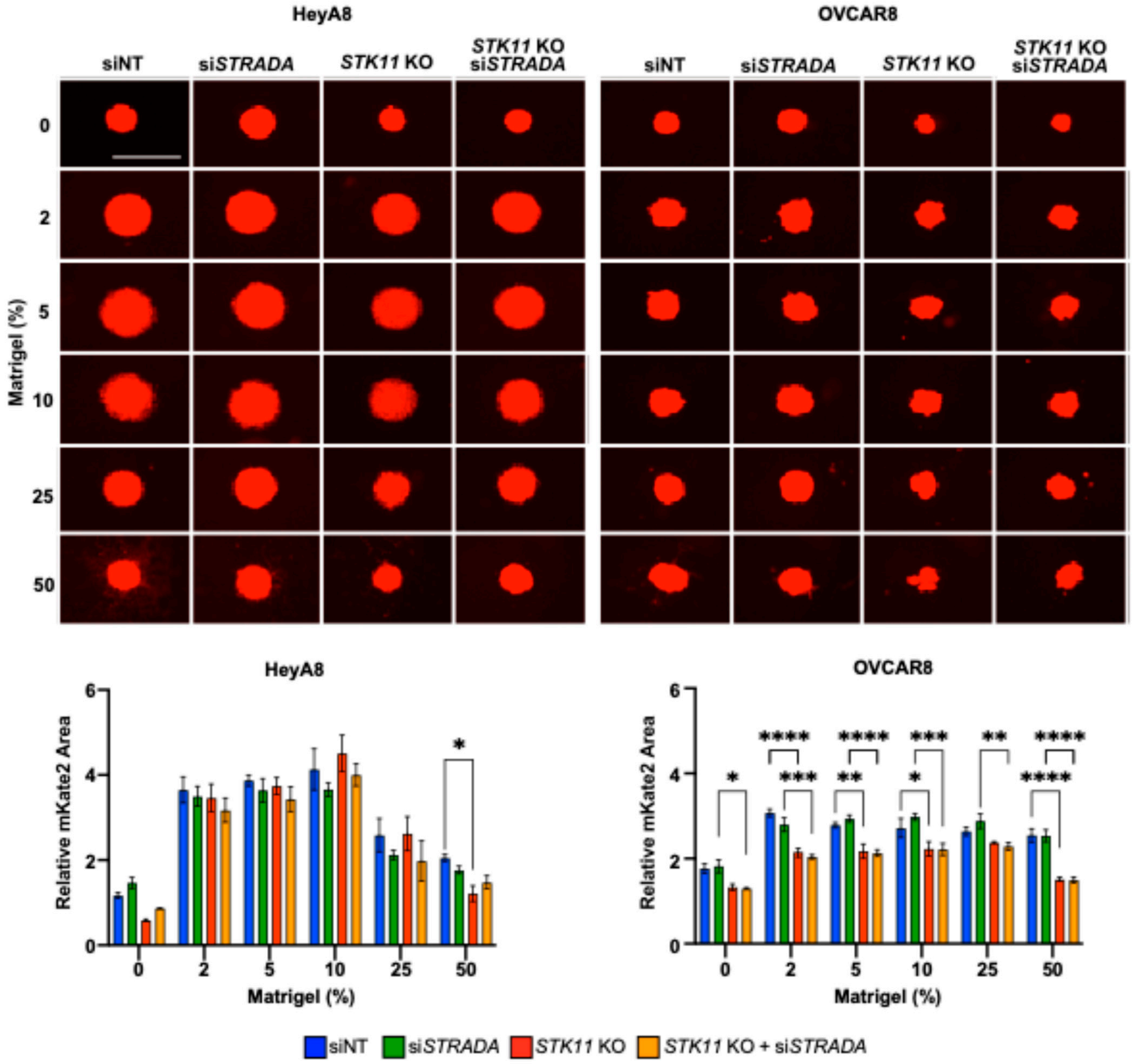

Supplemental Figure S7: Impact of LKB1 and STRADα loss on EOC invasion through Matrigel is dependent on matrix concentration.

HeyA8, HeyA8 *STK11* KO, OVCAR8, and OVCAR8 *STK11* KO cells expressing mKate2-NLS (red) were transfected with 10 nM siNT or 10 nM si*STRADA* for 72 h. After seeding 2,000 cells per well in a 96-well ULA plate for 24 h, spheroids were embedded in Matrigel at final concentrations of 0, 2, 5, 10, 25, or 50%, as indicated. After 72 h, the mKate2-NLS area was quantified using ImageJ from three independent experiments (mean ± SD) and graphed below the representative micrographs. Significance is indicated as * = P<0.05, ** = P<0.01, *** = P<0.001, and ****=P<0.0001. Scale bars = 1 mm.
